# Finite-size effects in transcript sequencing count distribution: its power-law correction necessarily precedes downstream normalization and comparative analysis

**DOI:** 10.1186/s13062-018-0204-y

**Published:** 2018-02-12

**Authors:** Wing-Cheong Wong, Hong-kiat Ng, Erwin Tantoso, Richie Soong, Frank Eisenhaber

**Affiliations:** 1 Bioinformatics Institute (BII), Agency for Science, Technology and Research (A*STAR), 30 Biopolis Street, #07-01, Matrix, Singapore, 138671 Singapore; 2 Cancer Science Institute of Singapore, National University of Singapore, Singapore, Singapore; 3 School of Computer Engineering (SCE), Nanyang Technological University (NTU), 50 Nanyang Drive, Singapore, 637553 Singapore

**Keywords:** Finite-size effects, Nyquist sampling criterion, Aliasing noise, Pareto distribution, Zip’s law, Transcript abundance, Sequencing, Normalization, Heteroskedasticity

## Abstract

**Background:**

Though earlier works on modelling transcript abundance from vertebrates to lower eukaroytes have specifically singled out the Zip’s law, the observed distributions often deviate from a single power-law slope. In hindsight, while power-laws of critical phenomena are derived asymptotically under the conditions of infinite observations, real world observations are finite where the finite-size effects will set in to force a power-law distribution into an exponential decay and consequently, manifests as a curvature (i.e.*, varying exponent values*) in a log-log plot. If transcript abundance is truly power-law distributed, the varying exponent signifies changing mathematical moments (e.g.*, mean, variance*) and creates heteroskedasticity which compromises statistical rigor in analysis. The impact of this deviation from the asymptotic power-law on sequencing count data has never truly been examined and quantified.

**Results:**

The anecdotal description of transcript abundance being almost Zipf’s law-like distributed can be conceptualized as the imperfect mathematical rendition of the Pareto power-law distribution when subjected to the finite-size effects in the real world; This is regardless of the advancement in sequencing technology since sampling is finite in practice. Our conceptualization agrees well with our empirical analysis of two modern day NGS (*Next-generation sequencing*) datasets: an in-house generated dilution miRNA study of two gastric cancer cell lines (*NUGC3 and AGS*) and a publicly available spike-in miRNA data; Firstly, the finite-size effects causes the deviations of sequencing count data from Zipf’s law and issues of reproducibility in sequencing experiments. Secondly, it manifests as heteroskedasticity among experimental replicates to bring about statistical woes. Surprisingly, a straightforward power-law correction that restores the distribution distortion to a single exponent value can dramatically reduce data heteroskedasticity to invoke an instant increase in signal-to-noise ratio by 50% and the statistical/detection sensitivity by as high as 30% regardless of the downstream mapping and normalization methods. Most importantly, the power-law correction improves concordance in significant calls among different normalization methods of a data series averagely by 22%. When presented with a higher sequence depth (*4 times difference*), the improvement in concordance is asymmetrical (*32% for the higher sequencing depth instance* versus *13% for the lower instance*) and demonstrates that the simple power-law correction can increase significant detection with higher sequencing depths. Finally, the correction dramatically enhances the statistical conclusions and eludes the metastasis potential of the NUGC3 cell line against AGS of our dilution analysis.

**Conclusions:**

The finite-size effects due to undersampling generally plagues transcript count data with reproducibility issues but can be minimized through a simple power-law correction of the count distribution. This distribution correction has direct implication on the biological interpretation of the study and the rigor of the scientific findings.

**Reviewers:**

This article was reviewed by Oliviero Carugo, Thomas Dandekar and Sandor Pongor.

**Electronic supplementary material:**

The online version of this article (10.1186/s13062-018-0204-y) contains supplementary material, which is available to authorized users.

## Author summary

In the grand scheme of things, the fundamental issue of reproducibility has a long-term implication on scientific rigor in this fast-paced OMICS-frenzy era. Since technology is not always WYSIWYG (*What you see is what you get*), it is important to validate our observations against some theoretical basis. For transcriptomic analysis, the lack of reproducibility is often hinted by the high discordance among normalization methods in a typical comparative analysis workflow given the same data set. Since important conclusions are often made based on these NGS-derived exploratory results, improving the reproducibility of the sequencing outputs becomes instrumental and ever more so since most bioinformatics analysis seldom bridge the gap between the exploratory finds and the true molecular actuators via the formal arguments of underlying molecular mechanisms. The latter is especially critical for clinical diagnostics applications.

## Background

Despite some cautionary notes on the generalization of power-law on natural phenomena [1], cell transcript abundance has often been theorized as originating from the family of power-law distributions [2]. Typically visualized in terms of histogram or rank-frequency plot, transcript abundance distribution seems to follow the extreme value theory where only a couple of genes are highly-expressed while the rest are relatively lowly-expressed. Earlier works on modelling SAGE-derived (*serial analysis of gene expression*) transcript abundance from vertebrates to lower eukaroytes have specifically singled out the power-law distribution, namely Zip’s law [3–7] where the slope of a power-law equation is about − 1 on a log-log scale. Originating from information theory, this slope describes the ideal compromise between the sender and receiver as the “Principle of Least Effort”; steep line represents a smaller and repetitive vocabulary while a shallower slope represents a larger and more diverse vocabulary. As such, Zipf statistic evaluates the balance between redundancy and diversity. Remarkably, Zipf’s law seemingly holds for most normal tissues of homogenous cell type and also approximately for the heterogenous cell type (i.e.*, the slope tends to be slightly lower than 1.0*) [4]. However, there exists a caveat to the power-law association: the observed power-law distribution of transcript abundance is usually imperfect in that it deviates from a single parameterized power-law slope.

By far, it has been unclear if this deviation is either reflective of the underlying true distribution or indicative of some inherent biases in terms of library size/sequencing depth [8], transcript lengths [9] and GC contents [10] in the physical or technological process that generates the observations. In our best understanding, the implications of the power-law deviation in transcript abundance has never been truly examined in current literature. Presumably, most researchers deem this deviation to have minimal effects on the downstream pre-processing steps like read mapping, normalization and statistical analysis. However, it is clear that there is no general consensus on the pre-processing of RNA-based sequencing data but rather best practices [11], with the normalization step contributing to the largest variation in the workflow performance [12–14].

In retrospect, all power-laws of critical phenomena are derived asymptotically under the conditions of infinite observations. In the real world, observations are finite and, therefore, the deviations from asymptotic power-law is to be expected in finite systems. The latter, which is known as finite-size effects, will force an observed power-law distribution into an exponential decay and presents itself as a curvature in the log-log plot [15]. Pertaining to the nature system that governs the cell transcript abundance, the critical question is to clarify if the observed power-law deviation is truly the result of the finite-size effects and not because the underlying distribution cannot be simply described by power-law [16, 17].

The implication here is that if transcript abundance is truly power-law distributed, its deviation or curvature on the log-log plot translates to varying exponent values which, in turn, signifies the changing mathematical moments (i.e.*, mean, variance, skewness, kurtosis*) of the distribution. Overall, this will manifest as heteroskedasticity (i.e.*, unequal variance within the data*) among the experimental replicates. Heteroskedasticity brings about two issues: Firstly, it introduces both bias and unequal variance to the data and poses additional difficulty to normalization methods where a good method aims to reduce variance without increasing bias [18]. Secondly, heteroskedasticity will bias test statistics since Type I and Type II error increases with underestimated and overestimated standard errors respectively as a consequence of unequal variance [19, 20].

In this work, we derived a generalized concept whereby the anecdotal description that transcript abundance sequencing count data is almost Zipf’s law-like distributed can now be objectively quantified by the Pareto power-law distribution via its mathematical moments and how the distribution can be rendered mathematically imperfect when subjected to the finite-size effects in the real world; a manifestation of the aliasing noise when undersampling occurs. Our formalism concurs well with our empirical analysis of two modern day NGS (*Next-generation sequencing*) datasets: an in-house generated dilution miRNA study of two gastric cancer cell lines (*NUGC3 and AGS*) and a publicly available spike-in miRNA data; Firstly, the finite-size effects causes deviations of sequencing count data from Zipf’s law and the issues of reproducibility issues in sequencing experiments that seems inescapable despite the advancement in sequencing technology since sampling is finite in the real world. Secondly, finite-size effects manifests as heteroskedasticity among experimental replicates to create statistical woes.

Collectively, this justifies for a simple restoration of the sequencing count data towards a power-law distribution with a single exponent value, herein as power-law correction, to reduce sample variance of lower transcript counts towards homoskedasticity for improved statistical outcomes. When this method was evaluated in a typical NGS comparative analysis workflow that entails (i) read mapping/count quantification (ii) pre-filtering of the zero counts across conditions (iii) normalization and (iv) the statistical testing, the signal-to-noise ratio (*SNR*) of the workflow improved by 50% after power-law correction. In turn, this higher SNR translates to an increase in statistical and detection sensitivity by approximately 30% in the dilution analysis regardless of the mapping and normalization methods used in the evaluation. Most importantly, the power-law correction addresses a long-standing issue of discordance in the comparative analysis workflow, particularly attributed to the variations among different normalization methods [12–14]. Using the dilution study, the increase in concordance rate was averagely 22% from the baseline rate of (48.24 ± 7.07)% to (70.32 ± 6.72)% upon power-law correction. When a higher sequencing depth is presented, power-law correction can extract the additional information content to increase significant detection. Specifically, in the dilution analysis, the higher sequencing depth instance (*by four times higher*) has an increase concordance rate of 32% (44.6% ± 4.91% versus 76.25% ± 1.78%) while it was 13% (51.88% ± 7.26% versus 64.39% ± 3.65%) for the lower depth instance. Finally, power-law correction statistically enhances the biological context of the experiment and elucidates the multiple metastatic signatures of the NUGC3 cell line in the dilution study of two gastric cell lines.

## Results and discussion

### Finite-size effects introduces curvature in sequencing count data distributions, impacts the reproducibility of the experiment and brings about heteroskedasticity among experimental replicates

Two miRNA sequencing datasets composed of technical replicates were being examined; The choice of miRNA is deliberate to avoid both transcript length bias [9] and abundance quantification [21] as confounding factors. The first miRNA set is the background count data of a spike-in experiment from a published study (*GEO dataset: GSE67074*) that contains 12 replicates [11]; The original authors’ BWA-mapped counts were used. The second set is an in-house generated dilution series of two gastric cancer cell lines - AGS and NUGC3 (*See methods for details: The dilution dataset* [22]). In this section, only the Bowtie1-mapped NUGC3 set of 8 technical replicates that spans across 4 concentration points of 12pM, 6pM, 3pM and 1.5pM was used. The varying concentration design aims to simulate the different sequencing depth (i.e.*, the total mapped reads*) that mimics a system of various sizes to study its finite-size effects (*See* Additional file 1: Figure S1).

Given that these datasets are made up of replicates, a simple intra-sample scaling where the counts of each replicate is divided by the maximum count of the same transcript within the replicate, will suffice. Furthermore, instead of visualizing Zipf’s law distribution with rank-frequency graphs, the Pareto distribution plots were used (*See methods for details: Transformation between rank-frequency and Pareto distribution*). This has the added advantage of characterizing the sequencing count data with the mathematical moments (i.e.*, mean, standard deviations*) of the Pareto’s density function that is lacking in a typical Zip’s law plot.

**Fig. 1:**
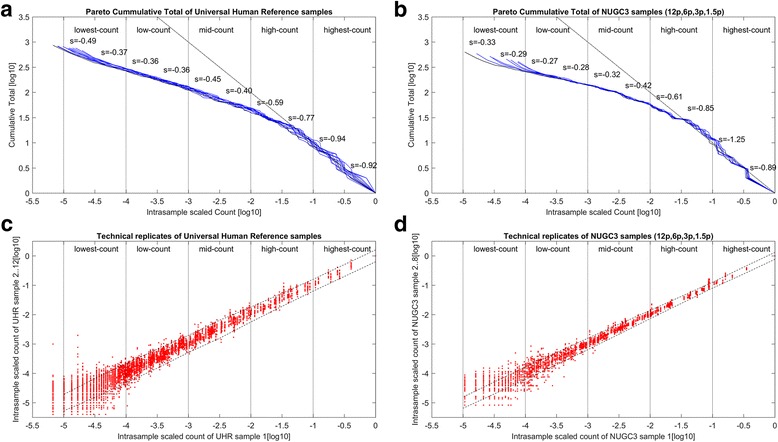
Pareto distributions and scatterplots of spike-in background and dilution datasets. **a** and **b** give the Pareto distribution plots of the scaled background counts from the spike-in background and NUGC3 dilution dataset respectively. Both plots are segmented into the highest-count to lowest-count regions based on an order of magnitude per segment (*see vertical dotted lines across horizontal axis*). Generally, Zipf’s law (i.e.*, slope of − 1*) holds well for the highest-count segments. **c** and **d** give the scatterplots of the highest sequencing depth replicate against the rest for the spike-in background and NUGC3 dilution dataset respectively. Both plots exhibit the hallmark of the Pareto’s mathematical moments where a change in variance is perpetuated by a change in the power-law exponent. The noise that plagued the low and lowest-count segments, serves to highlight the instability of the replicated count values when the corresponding power-law mathematical moments stem not only from low exponent values but of non-comparable magnitude as well

Figure 1a and b depict the cumulative histograms, specifically the Pareto distribution plots of the scaled counts from the spike-in background and NUGC3 dilution dataset (*See methods for details: Property of Type I Pareto distribution*). The plots are segmented into its appropriate highest-count to lowest-count linear ranges based on an order of magnitude per segment (*see vertical dotted lines across horizontal axis*). In both cases, the highest-count segments approach the Zipf’s law (*see dashed black line*) which has a characteristic slope of − 1. Beyond that, the slope values generally decreased and finished with an inflection for the lowest-count segments. While there is a general convergence of slope values from the highest-count to the mid-count segments, a specific divergence for the low and lowest-count segments can be readily seen. In the case of the dilution set, its divergence is more exaggerated (*as marked by the split-end pattern*) as a consequence of a more deliberate sequencing depth differences among the replicates. The latter marks the effects of the finite-size effects which plays a major role in the reproducibility of the observed distributions.

Meanwhile, the trend towards changing slopes along the count segments indicates a general deviation from a single power-law exponent. Based on the mathematical moments of the Pareto distribution (Eqs. 3 and 4), exponent values of below “1” indicates asymptotically infinite moments. The consequence of these infinite moments is that their empirical estimates can converge very slowly due to the frequent occurrences of extreme values [23]. When coupled with the changing exponents along the count segments, heteroskedasticity (i.e.*, unequal error variance*) among the replicates can be expected from the imperfect power-law distributions.

To further emphasize, the scatterplots of the scaled counts for the 11 replicates of the spike-in background set against the replicate with the highest total reads were examined in Fig. 1c. Concurrently, Fig. 1d depicts the scaled count of the 7 NUGC3 replicates of the dilution set against the NUGC3 12pM sample with the highest total reads. Similar segmented ranges are also superimposed on these figures. Complementing Fig. 1c and d, the regression slope of the power-law fit, the total number of points, the observed and expected standard deviation of each segmented range were computed and complied in Table 1. Of particular importance is the expected standard deviation which projects the expected heteroskedasticity of the replicate noise across the count segments. It is extrapolated from the observed standard deviation of a reference count segment after accounting for the slope differences between the reference segment and the other segments (*See* Table 1 *legend for further explanation*).

**Table 1:**
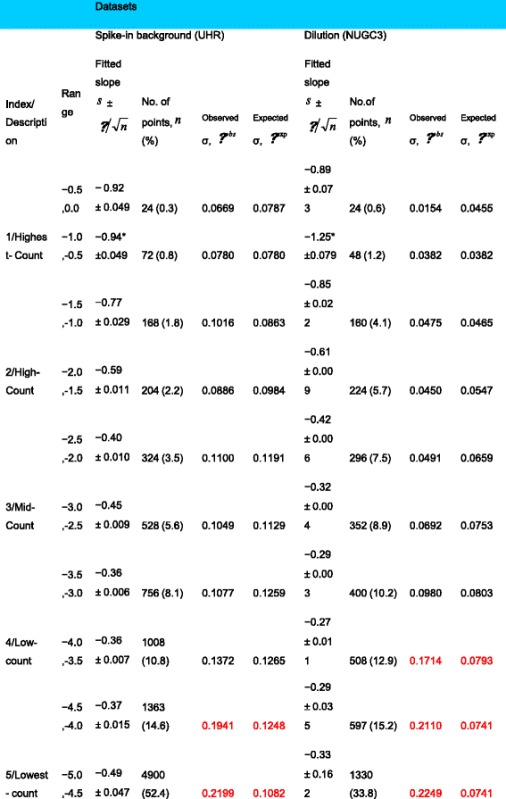
Summary of analysis for spike-in background and NUGC3 dilution datasets

The summarized analysis for two datasets, namely the spike-in background and dilution datasets, were presented. The spike-in set consists of 1387 transcripts over 12 replicates while the dilution set has 865 transcripts over 8 replicates. For each segmented range, the fitted slope to Pareto distribution, the total number of points, the observed and expected standard deviation are calculated. The expected standard deviation *σ*^exp^ gives the corrected standard deviation of each “slope < 1” segment as if its slope is the same as the reference segment (indicated by *). It is calculated via the formula ${\sigma}_{{\mathit{\operatorname{seg}}}_i}^{\mathrm{exp}}={\sigma}_{{\mathit{\operatorname{seg}}}_{ref}}^{obs}\left({s}_{{\mathit{\operatorname{seg}}}_{ref}}/{s}_{{\mathit{\operatorname{seg}}}_i}\right)$ using the highest-count segment as the reference. For the spike-in set, the observed and expected standard deviation is about 2 times larger while this is about 3 times for the dilution set (highlighted in red) in the worst case

Essentially, the observed heteroskedasticity seen in the Fig. 1c and d exhibits the hallmark of the Pareto’s mathematical moments where a change in variance is perpetuated by a change in the power-law exponent. Furthermore, the observed heteroskedasticity can be divided into variances of reproducible (i.e.*, the degree of agreement between experimental results conducted by different individuals/locations/instruments*) and irreproducible origin. Specifically, when heteroskedasticity is about equal between the observed (i.e.*, general spread of the datapoints*) and the expected (i.e.*, margins marked by the dotted lines at 99% confidence interval*) standard deviations, it is simply reflective of the reproducible replicate noise as for the cases of the highest to mid-count segments. However, when heteroskedasticity spreads beyond the expected margins, it indicates additional irreproducible noise as for the cases of the diverged low and lowest-count segments. In the worst cases, the observed standard deviation exceeds that of the expected by about 2 times for the spike-in background set and 3 times for the NUGC3 dilution set (*See* Table 1*: values in red*).

The irreproducible noise that plagued the diverging low and lowest-count segments, serves to highlight the instability of the replicated count values when the corresponding power-law mathematical moments stem not only from low exponent values but of non-comparable magnitude as well. The latter basically demonstrates the impact of the finite-size effects on the same physical system when sampled at different rates. Since irreproducibility can occur even for a set of replicates that has similar sequencing depths like the case of the spike-in set, it is expected to be worse for any datasets that have more diverse depths as attested by the dilution set.

Unfortunately, none of the commonly used normalization methods namely DESeq [24, 25], Relative Log Expression (RLE) [24, 26], Trimmed Mean of M-values (TMM) [26, 27], UpperQuartile (UQ) [12, 26], Count Per Million (CPM) [26] and Quantile [18, 28]) can correct for the power-law deviations in both datasets; Both power-law deviation and heteroskedasticity remain (*See* Additional files 2: Figure S2 and Additional files 3: Figure S3).

### Aliasing noise explains the finite-size effects that distorts the theoretical power-law distribution of sequencing count data

In fact, the sequencing procedure can be recast into a sampling problem: The total transcript population in a cell can be viewed as a library of unique transcript species with different frequency of occurrences. Simply put, this library can be thought as the composites of a continuous analogue signal. And when this analogue signal is subjected to sequencing, it undergoes a sampling procedure where the abundance of the individual transcript species in terms of its counts, is being quantified. Collectively, the digitized counts becomes the sampled signal of the original analogue signal.

Mathematically, a power-law type sampled signal *Y*(*f*) with an amplitude of *S*_*o*_ and an exponent of *α*, can be described as the sum of its original signal *S*_*o*_*f*^−*α*^ and its alias term *S*_*o*_(*f*_*s*_ − *f*)^−*α*^ given any frequency *f* (see Eq. 13) and can be visualized as a frequency-domain plot. With any sampling procedure, undersampling will occur when the Nyquist sampling criterion of *f*_max_ < 2*f*_*s*_ is not satisfied where *f*_max_ is the largest frequency component of the original signal and *f*_*s*_ is the sampling frequency. As a consequence, this will result in a non-zero alias term *S*_*o*_(*f*_*s*_ − *f*)^−*α*^. More specifically, the condition of aliasing where a distortion of the sampled signal *Y*(*f*) from its original signal will occur [29] (*See methods for details: Derivation of the alias term in the power-law* 1/*f*^*α*^*equation;*

*Eqs. 5-13*).

In relation to the sampled signal *Y*(*f*), the rank variable *y* and maximum count value *C*_1_ of the rank-frequency equation (*see Eq. 14*) are analogous to the frequency *f* and the amplitude *S*_*o*_ of *Y*(*f*) respectively. In turn, the rank-frequency and Pareto’s tail distribution are inversely related to each other (*See methods for details: Transformation between rank-frequency and Pareto distribution; Eqs. 14-17*). Essentially, the Pareto plots can be straightforwardly transformed into a frequency-domain plot.

To determine if undersampling has occurred, the sampling frequency *f*_*s*_ needs to be first determined between the sampled signal and its original signal to check if the Nyquist sampling criterion is fulfilled. The best estimate or surrogate of the original signal *S*_*o*_*f*^−*α*^ can be estimated from the replicate with the largest total reads within the data series. For the dilution set, this was one of the 12p NUGC3 sample which consists of a total of 632 unique count values. In the case of the spike-in background set, the replicate with the largest total reads has 863 unique count values. Corresponding to their rank-frequency (*frequency-amplitude*) plots, this translate to a maximum rank (*frequency*) of 632 and 863 accordingly.

Using the respective surrogates as baseline, the observed alias noise between a sampled signal and its original signal can be then determined by taking their logarithmic differences as described by the mathematical expression logΔ*Y*(*f*) = log[*S*_*o*_*f*^−*α*^ + *S*_*o*_(*f*_*s*_ − *f*)^−*α*^] − log[*S*_*o*_*f*^−*α*^] (*see Eq. 19*). Since Zip’s law (*see eq. 14 where b* = 1) holds for the high and highest-count segments of both datasets, the exponent term is implicitly set to *α* = 1. Alias noise Δ*Y*(*f*) reaches its maximum when *f* = *f*_max_ such that Δ*Y*(*f*) = Δ*Y*(*f*_max_), for which the sampling frequency *f*_*s*_ can be solved by evaluating logΔ*Y*(*f*_max_) (*See methods for details: Solving for sampling frequency f*_*s*_*to determine undersampling; Eqs. 18–21*).

**Fig. 2:**
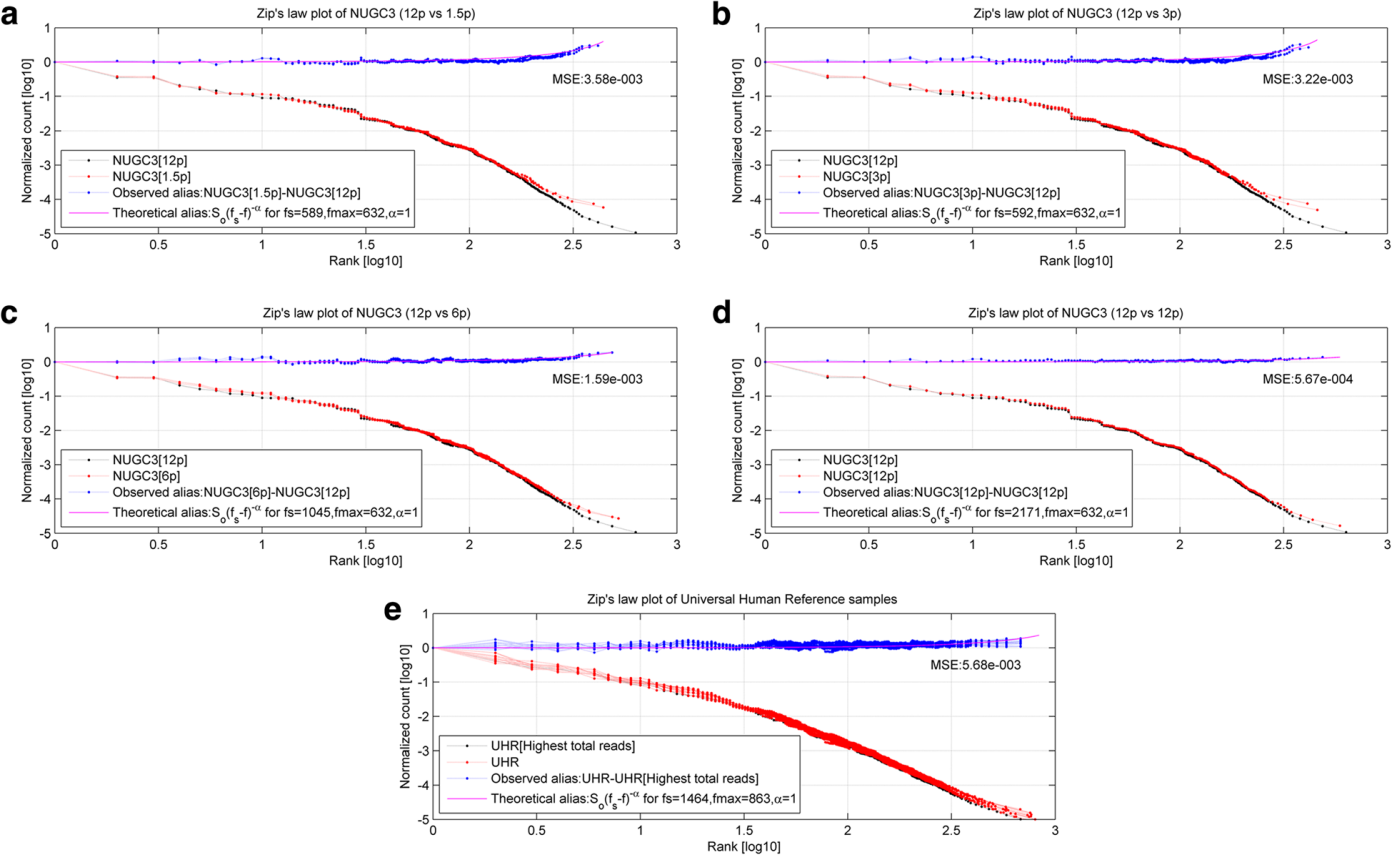
Rank-frequency plots of NUGC3 dilution and spike-in background datasets. **a**, **b**, **c**, **d** and **e** show the rank-frequency plots for the 1.5p pair, 3p pair, 6p pair, single 12p replicate and the 11 UHR replicates against the best estimate of the original signals (*marked in black*). Meanwhile, the observed alias noise (*marked in blue*) and the theoretical alias noise *S*_*o*_(*f*_*s*_ − *f*)^−*α*^ (*marked in magenta*), are also shown. In each subplot, the sampling frequency *f*_*s*_ and the mean square error (*MSE is defined as the residual error between the observed and theoretical alias noise*) are given as well. Overall, the low MSE values of between 5.67e-4 to 3.58e-3 indicates a good fit between the theoretical alias noise model and the observed alias datapoints. For the NUGC3 dilution set, the 1.5p, 3p, 6p replicates have failed to satisfy the Nyquist sampling criterion of *f*_max_ < 2*f*_*s*_ at sampling frequencies of 589, 592 and 1045; Undersampling has occurred for these cases. The same can also be concluded for the spike-in background dataset. Only the single 12p case had satisfied the Nyquist criterion at *f*_max_ < 3.4*f*_*s*_

Furthering the analysis of the scaled datasets in Fig. 1, Fig. 2 shows the rank-frequency plots for the NUGC3 dilution and the spike-in replicates (*marked in red*). In particular, Fig. 2a–e show the plots for the 1.5p pair, 3p pair, 6p pair, single 12p replicate and the 11 UHR replicates against the best estimate of the original signals (*marked in black*). In addition, the observed alias noise (*marked in blue*), together with the corresponding theoretical alias noise *S*_*o*_(*f*_*s*_ − *f*)^−*α*^ (*marked in magenta*), are shown in the sub-figures. For each case, the sampling frequency *f*_*s*_ and the mean square error (*MSE is defined as the residual error between the observed and theoretical alias noise*) are given as well. The overall low MSE values of between 5.67e-4 to 3.58e-3 indicates a good fit between the theoretical alias noise model and the observed alias datapoints.

Within the NUGC3 dilution set, the 1.5pM, 3pM, 6pM replicates have failed to satisfy the Nyquist sampling criterion of *f*_max_ < 2*f*_*s*_ at sampling frequencies of 589, 592 and 1045 (*See* Fig. 2a–c) respectively. Since the minimum sampling frequency needed by the NUGC3 dilution set is 1264 (2 × 632), undersampling has occurred for these cases. Undersampling can also be concluded for the spike-in background dataset at a sampling frequency of 1464 (*See* Fig. 2e) where the required minimum sampling frequency is 1726 (2 × 863). In contrast, only the single 12pM case had satisfied the Nyquist criterion at *f*_max_ < 3.4*f*_*s*_ (*See* Fig. 2d). Theoretically, the sampling frequency for a zero alias noise tends to infinity (*solve eq. 17 for* Δ*Y*(*f*_max_) = 1 *at f* = *f*_max_).

In hindsight, the finite-size effects has always plagued sequencing-based studies since the early days [7] where the alias noise manifests as the misfitted tail in Zipf’s law distributions. The magnitude of the finite-size effects is dependent on the severity of undersampling and it can now be quantified formally through a simple recasting of the Pareto plot to the frequency-domain plot.

### The necessity of power-law correction on sequencing count data to restore distribution distortion

**Fig. 3:**
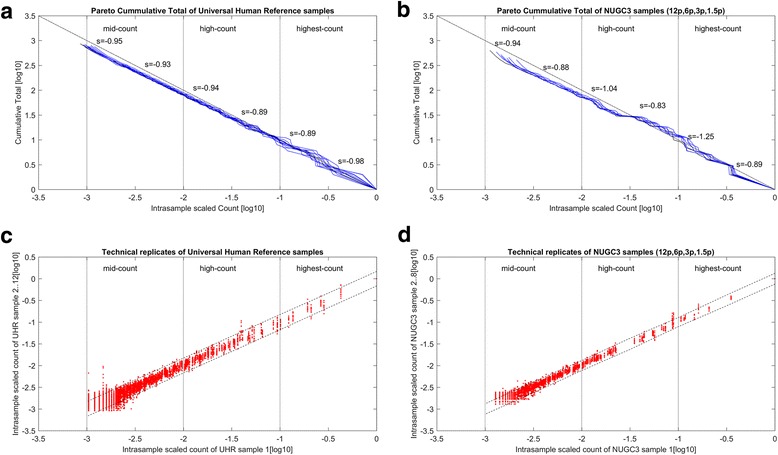
Post power-law correction, Pareto distributions and scatterplots of spike-in background and dilution datasets. **a** and **b** give the Pareto distribution plots of the scaled background counts from the spike-in background and NUGC3 dilution dataset respectively, after the power-law correction was applied. Both plots are segmented into the highest-count to lowest-count regions based on an order of magnitude per segment (*see vertical dotted lines across horizontal axis*). Both plots display a power-law distribution with a more uniform slope throughout all count segments. In fact, the power-law correction estimates how the true underlying distribution should have been without aliasing. Meanwhile, **c** and **d** show that the corrected count values exhibit less heteroskedasticity across all count segments and variation among the replicates with the increase in slope values after the power-law correction. Finally, the minimum count value of each replicate has increased such that the uncorrected count values previously (*See* Fig. 2C and D) in the low and lowest-count segment have now been moved into the mid-count segment

The restoration of the power-law plots towards a common power-law slope were applied to the NUGC3 dilution and spike-in background data series. (*See methods for details: Computation procedures for power-law correction of a count data set*). Akin to Figs. 1 and 3 shows the Pareto plots and scatterplots of both the power-corrected spike-in background and the NUGC3 dilution datasets with the same intra-sample scaling applied. Table 2 complements Fig. 3 with the details on the regression slope of the power-law fit, the total number of points, the observed and expected standard deviation of each segmented range.

**Table 2:**
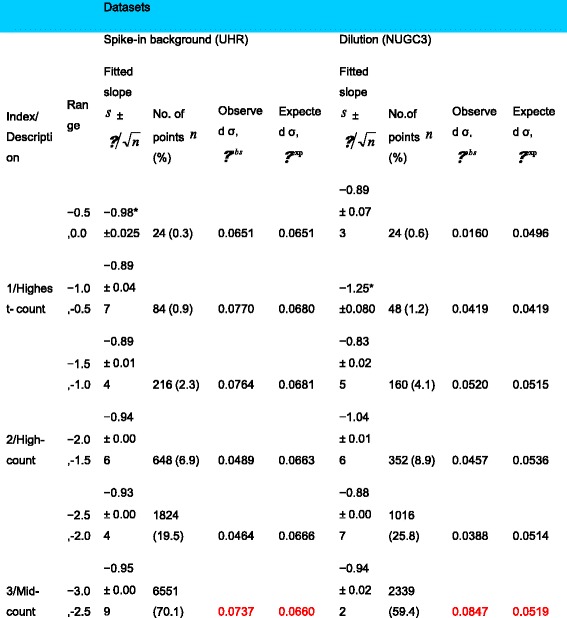
Summary of analysis for the power-law corrected spike-in background and NUGC3 dilution datasets

The summarized analysis of the Zipf’s law corrected datasets, namely the spike-in background and dilution datasets, were presented. The spike-in set consists of 1387 transcripts over 12 replicates while the dilution set has 865 transcripts over 8 replicates. For each segmented range, the fitted slope to Pareto distribution, the total number of points, the observed and expected standard deviation are calculated. The expected standard deviation *σ*^exp^ gives the corrected standard deviation of each “slope < 1” segment as if its slope is the same as the reference segment (indicated by *). It is calculated via the formula ${\sigma}_{{\mathit{\operatorname{seg}}}_i}^{\mathrm{exp}}={\sigma}_{{\mathit{\operatorname{seg}}}_{ref}}^{obs}\left({s}_{{\mathit{\operatorname{seg}}}_{ref}}/{s}_{{\mathit{\operatorname{seg}}}_i}\right)$ using the highest-count segment as the reference. For the spike-in set, the observed and expected standard deviation is about 1.1 times larger while this is about 1.6 times for the dilution set (highlighted in red) in the worst case

Generally speaking, the Pareto plots in both Fig. 3a and b show a power-law distribution with a more uniform slope throughout all count segments, which averages to about − 0.94 (see Table 2 column 3) for the spike-in background data set and − 0.97 (see Table 2 column 7) for the NUGC3 dilution data set. The restoration to a single exponent of the Pareto plot through the power-law correction gives us an estimate of how the true underlying distribution (*see dashed line that depicts the Zipf’s law distribution*) would have looked if there had been no aliasing issues.

With larger slope values than before, it implies that the standard deviation for all count segments, should theoretically converge towards a smaller value. Indeed, Fig. 3c and d of the respective data sets show that the corrected count values exhibit less heteroskedasticity across all count segments and variation among the replicates. This reduced heteroskedasticity is to be expected if transcript abundance is power-law distributed and adheres to its mathematical moments (*see Eqs. 3 and 4*); In hindsight, it does indeed. Furthermore, based on Table 2 (*markings in red*), the difference between the observed and expected standard deviation is merely 1.1 times for the spike-in background dataset and 1.6 times for the NUGC3 dilution dataset in the worst case. The stark improvement from before the power-law correction (i.e.*, worst case of 2 times and 3 times respectively*) signifies that the irreproducible noise in the data series has been dramatically reduced in the form of alias noise. Overall, it translates to a smaller dynamic range for the corrected values where the uncorrected count values from the low and lowest-count segment have now been shifted to the mid-count segment.

**Fig. 4:**
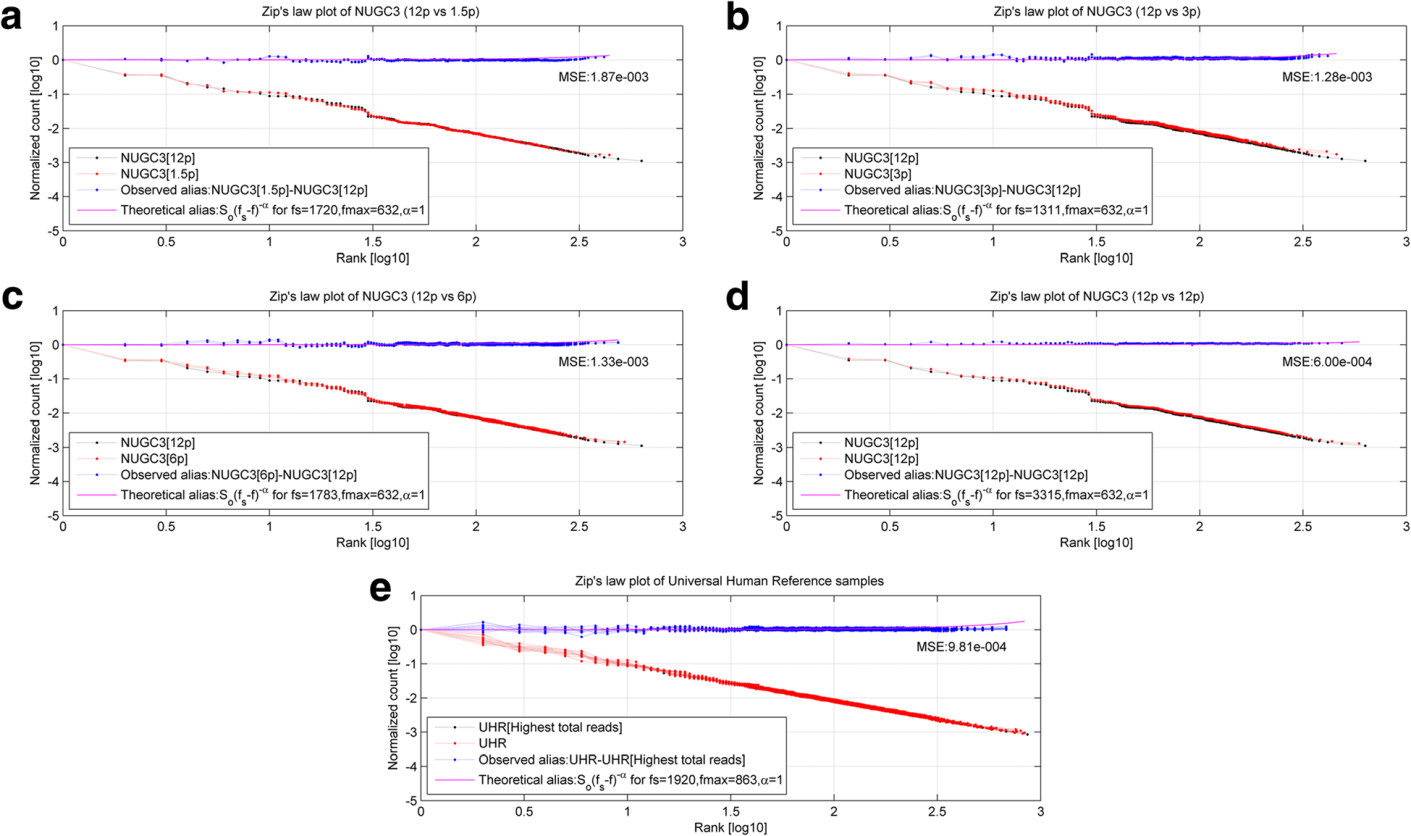
Post power-law correction, rank-frequency plots of NUGC3 dilution and spike-in background datasets. **a**, **b**, **c**, **d** and **e** show the rank-frequency plots for the 1.5p pair, 3p pair, 6p pair, single 12p replicate and the 11 UHR replicates against the best estimate of the original signals (*marked in black*) after the power-law correction. The observed alias noise (*marked in blue*) and the theoretical alias noise *S*_*o*_(*f*_*s*_ − *f*)^−*α*^ (*marked in magenta*), are also shown. In each subplot, the sampling frequency *f*_*s*_ and the mean square error (*MSE is defined as the residual error between the observed and theoretical alias noise*) are given as well. The overall low MSE values of between 6.00e-4 to 1.87e-3 indicates a good fit between the theoretical model and the observed alias. Generally speaking, the corrected datasets shows a general absence of undersampling. For all plots, the observed alias noise (*marked in blue*), as well as the theoretical alias noise *S*_*o*_(*f*_*s*_ − *f*)^−*α*^ (*marked in magenta*), shows very slight aliasing in all cases given their new sampling frequencies of 1720, 1311, 1783, 3315 and 1920 respectively

When the corrected spike-in background and NUGC3 dilution data sets were subjected to a re-analysis of aliasing, the corrected datasets shows a general absence of undersampling. The rank-frequency plots for the corrected dilution replicates are depicted by Fig. 4a for the 1.5p pair, Fig. 4b for the 3p pair, Fig. 4c for the 6p pair and Fig. 4d for the single 12p, while Fig. 4e shows the corrected spike-in background replicates for the set of 12 UHR replicates (*marked in red*). The best estimate of the original signal is marked by black in each figure. The corresponding observed alias noise (*marked in blue*), as well as the theoretical alias noise *S*_*o*_(*f*_*s*_ − *f*)^−*α*^ (*marked in magenta*), shows very slight aliasing in all cases given their new sampling frequencies of 1720, 1311, 1783, 3315 and 1920 respectively. The overall low MSE values of between 6.00e-4 to 1.87e-3 indicates a good fit between the theoretical model and the observed alias.

### Power-law correction should precede normalization; it increases signal-to-noise ratio and sensitivity of statistical testing/detection in comparative analysis

**Fig. 5:**
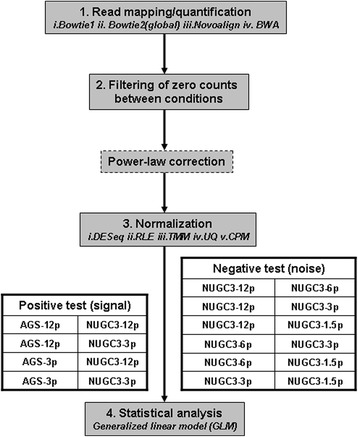
NGS comparative analysis evaluation workflow. The workflow broadly entails the following 4 steps: (i) the read mapping to produce transcript count, (ii) the filtering of the transcripts to ensure non-zero (i.e.*, no missing*) count values between conditions, (iii) the application of a normalization procedure to minimize both bias and variance and finally, (iv) the statistical testing to elucidate significant genes based on some pre-determined *p*-value and fold-change cutoff. As such, the correction step is best inserted after the filtering step and before the normalization step

To rigorously evaluate the impact on power-law correction in a typical NGS comparative analysis workflow, Fig. 5 shows the evaluation setup that permutes across 4 mapping algorithms (*Bowtie1, Bowtie2(global)* [30]*, Novoalign (**www.novocraft.com**) and BWA* [31, 32]) and 6 normalization methods (*DESeq* [24, 25]*, Relative Log Expression (RLE)* [24, 26]*, Trimmed Mean of M-values (TMM)* [26, 27]*, UpperQuartile (UQ)* [12, 26]*, Count Per Million (CPM)* [26] *and Quantile normalization* [18, 28]). Furthermore, the comparisons were split into the positive (*signal between NUGC3 and AGS samples*) and the negative (*noise within the NUGC3 replicates*) tests. For the statistical analysis, the generalized linear model [33] from the EdgeR package [26] was used for the multiple contrasts where each comparison produced a set of fold-change values, average counts (*in terms of counts-per-million*) and *p*-values (*See methods for details: Generalized NGS comparative analysis*).

**Fig. 6:**
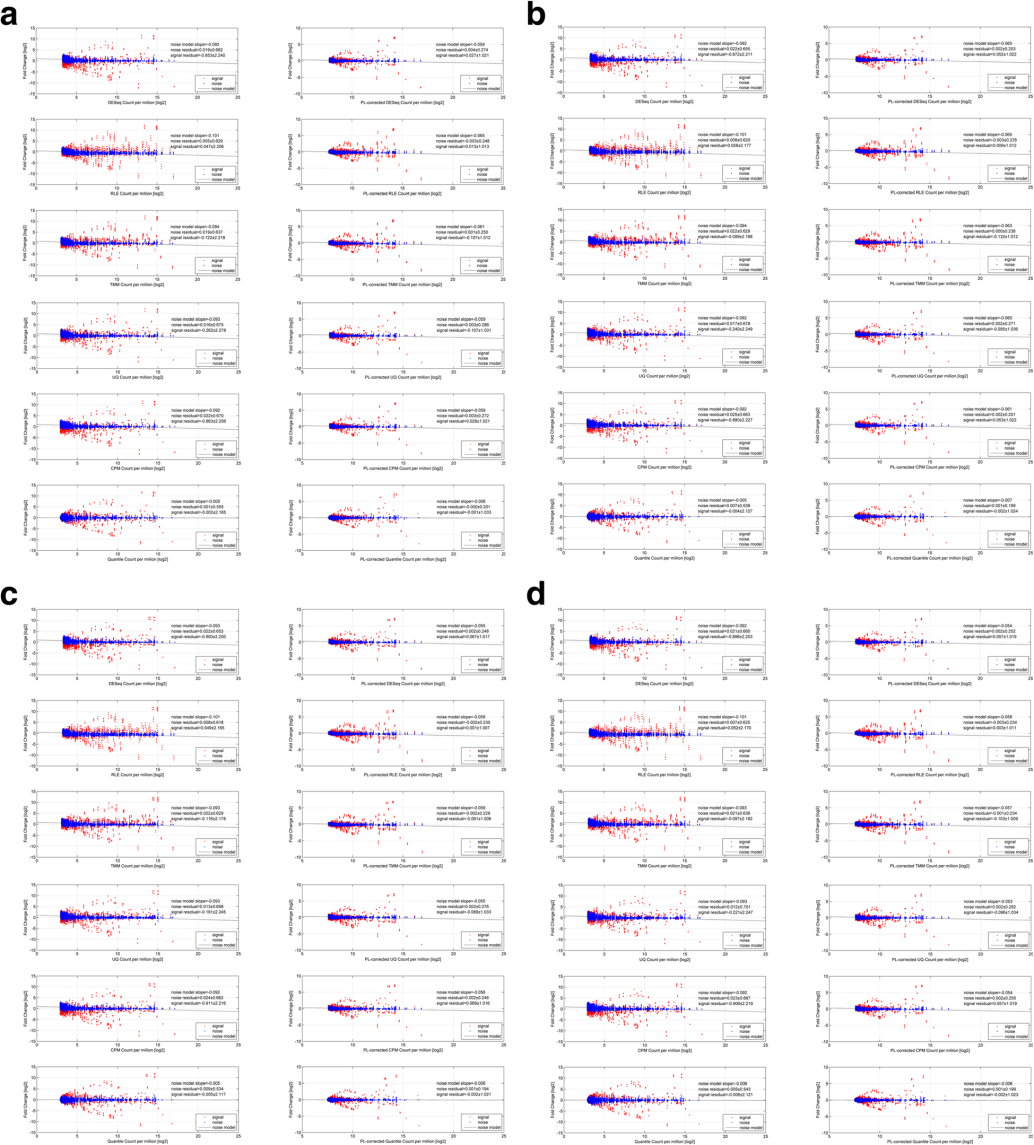
MA-plots of dilution data set before and after power-law correction. Fig. 6 shows the MA-plots (*i.,e., average counts* versus *fold-changes*) of the dilution dataset before (*left-column*) and after (*right-column*) the power-law correction. In particular, Figs **a**, **b**, **c** and **d** shows the MA-plot analysis for 4 mapping (*Bowtie1, Bowtie2(global), Novoalign and BWA*) algorithms while the permutation of the 6 normalization algorithms (*DESeq, Relative Log Expression (RLE), Trimmed Mean of M-values (TMM), UpperQuartile (UQ), Count Per Million (CPM) and Quantile normalization*) are arranged in a row-wise manner. For the power-law correction, the optimum PPS setting was evaluated to be 55 (*See* Additional file 6: Fig. S5A). In each MA-plot, the positive and noise signal are shown in red and blue respectively. The noise model (*y* = *mx*) is shown in dotted lines; Ideally, the slope value is 0 for no bias. The signal and noise residuals with respect to the noise model give the fold-change variation along the average count axis (*or x-axis*). Overall, it is apparent that the heteroskedasticity (*see left-column*) of the uncorrected AGS and NUGC3 count values has propagated down to the level of comparative analysis regardless of any combination of mapping and normalization methods. However when power-law correction is applied, heteroskedasticity was dramatically minimized (*see right-column*)

Figure 6a shows the MA-plots (*i,e., average count* versus *fold-change*) of the Bowtie1-mapped dilution dataset before (*left-column*) and after (*right-column*) the power-law correction for the 6 normalization algorithms (*arranged in row-wise*). This Bowtie1-mapped set comprises of 637 paired AGS-NUGC3 paired-transcripts. Likewise, Fig. 6b–d depict the MA-plots of the Bowtie2(global), Novoalign and BWA-mapped dilution analysis where the total amount of mapped transcripts are 657, 673 and 670 respectively. Their respective PPS settings was referenced from the Bowtie1-mapped set’s optimum setting to standardize the parameter settings of the power-law correction step across the mapping algorithms (*See methods for details: Computation procedures for power-law correction of a count data set*).

For each MA-plot, the positive signal is depicted in red while the noise is shown in blue. The noise model, as a simple linear regression of *y* = *mx*, attempts is depicted dotted line. For both signal and noise datapoints, their corresponding residual with respect to the fitted noise model gives the fold-change variation along the average count axis (*or x-axis*) and can be recapitulated into a summary statistics. Essentially, the summary statistics gives the amount of bias (*the mean*) and variance (*the standard deviation*) of the normalization method where an effective one should reduce variance without increasing bias [18]. Furthermore, signal-to-noise ratio (SNR) of each mapping/normalization pair, defined as $E\left({x}_{signal}^2\right)/{\sigma}_{noise}^2$ where $E\left({x}_{signal}^2\right)$ is the expectation of the second moment of the signal and ${\sigma}_{noise}^2$ is the variance of the noise, was also computed. For each mapping algorithm, the median measures of the signal residual, noise residual and SNR across all normalization methods are also taken and summarized in Table 3 (*see* Additional file 4: Table S1 for full details).

**Table 3:** The average signal-to-noise characteristics of the comparative dilution analysis (AGS versus NUGC3) before and after power-law correction

	Original data			Power-law corrected data		
Mapping method	Median residual (μ ± σ)_noise_	Median residual (μ ± σ)_signal_	Median signal-to-noise ratio $\frac{E\left({x}_{signal}^2\right)}{\sigma_{noise}^2}$	Median residual (μ ± σ)_noise_	Median residual (μ ± σ)_signal_	Median signal-to-noise ratio $\frac{E\left({x}_{signal}^2\right)}{\sigma_{noise}^2}$
Bowtie1	0.018 ± 0.649	−0.192 ± 2.229	11.3	0.002 ± 0.261	0.006 ± 1.021	15.4
Bowtie2 (global)	0.019 ± 0.642	−0.169 ± 2.200	11.3	0.002 ± 0.244	0.003 ± 1.022	17.6
Novoalign	0.017 ± 0.641	−0.153 ± 2.189	11.3	0.001 ± 0.238	−0.001 ± 1.017	18.2
BWA	0.017 ± 0.648	−0.159 ± 2.193	11.1	0.001 ± 0.242	0.001 ± 1.019	17.8

This table complements the MA-plots in Fig. 6A to D. It summarizes the characteristics of the signal and noise comparisons before and after power-law correction for each aligner across 6 normalization methods. The bias and variance of each normalization method, in terms of signal and noise, are computed from the difference between the comparisons and the fitted noise model and with the summary statistics taken. The signal-to-noise ratio, before and after power-law correction, are also given. The average signal-to-noise ratio improvement is about 1.5 times after the correction

Throughout all the MA-plots, heteroskedasticity in the noise comparisons (*depicted in blue*) can be readily seen without the power-law correction. Heteroskedasticity brings about two issues: Firstly, it introduces both bias and large variance to the comparisons as attested by the mean and standard deviation ranges of − 0.192 to − 0.153 and 2.189 to 2.229 for the positive comparisons (or signal) (Table 3 *column 3*). In contrast, this was between 0.001 to 0.006 and between 1.017 to 1.022 for power-law corrected analysis (Table 3 *column 6*). Overall, the correction improved the SNR by about 50% (i.e.*, 17–11/11*) given the SNR of the corrected and uncorrected analysis at about 17 times and 11 times respectively (Table 3 *columns 4 and 7*).

**Fig. 7:**
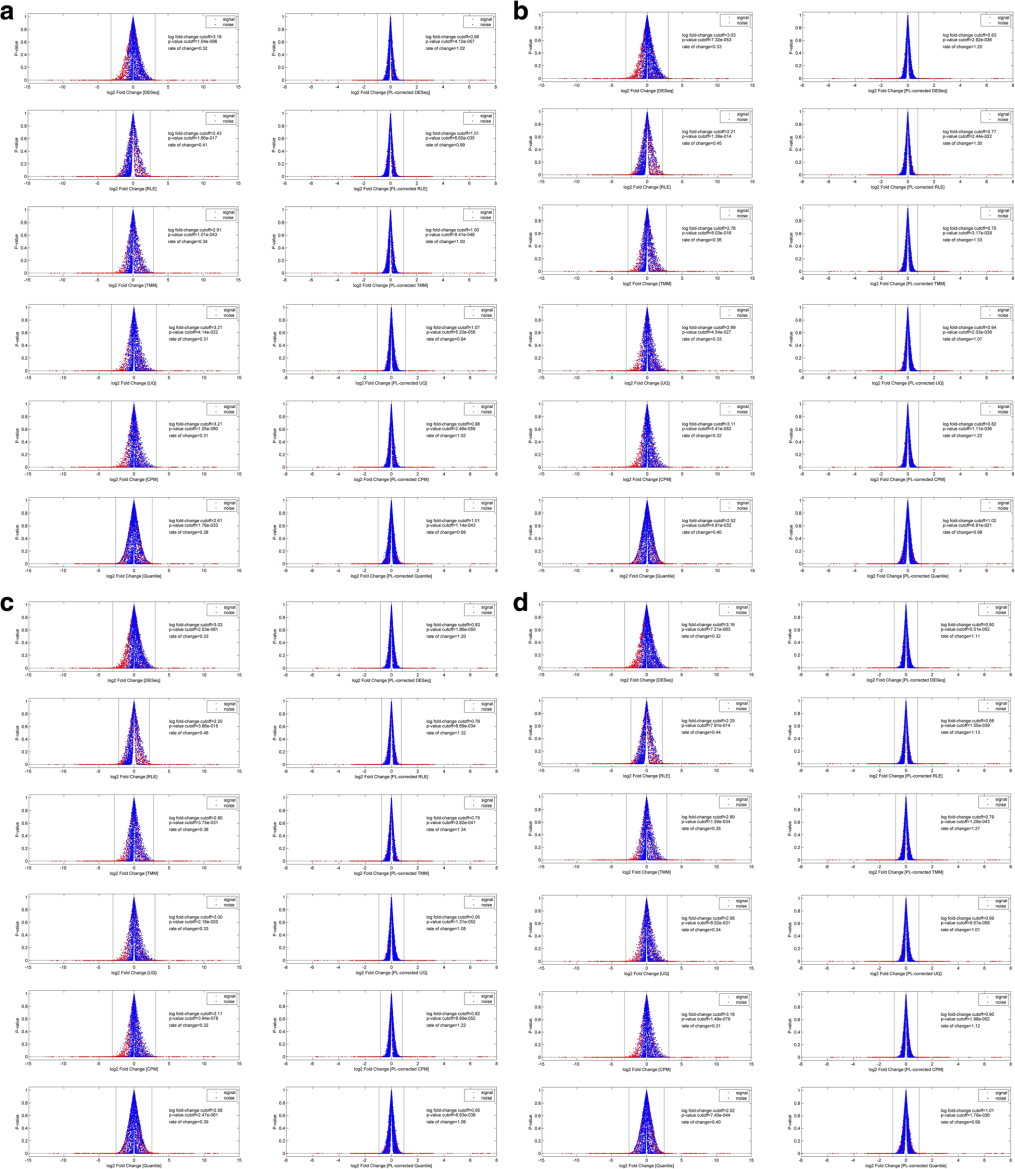
Volcano plots of dilution data set before and after power-law correction. Akin to Fig. 6, the volcano plots of the dilution dataset before (*left-column*) and after (*right-column*) the power-law correction is shown in Fig. 7. In particular, Figs **a**, **b**, **c** and **d** shows the MA-plot analysis for 4 mapping (*Bowtie1, Bowtie2(global), Novoalign and BWA*) algorithms while the permutation of the 6 normalization algorithms (*DESeq, Relative Log Expression (RLE), Trimmed Mean of M-values (TMM), UpperQuartile (UQ), Count Per Million (CPM) and Quantile normalization*) are arranged in a row-wise manner. Overall, the apparent asymmetrical spread of the noise comparisons (*in blue*) of the uncorrected data set demonstrates the non-zero fold-change bias despite the application of various normalization methods. Most importantly, the slower rate of change in *p*-values of the uncorrected cases (*see left-column*) when compared to the power-law corrected cases (*see right-column*), implies that a higher fold-change threshold is needed to acquire the same p-value (*or Type I error rate*) during statistical testing. In turn, a higher fold-change threshold also implies a larger type II error (i.e.*, failing to detect an effect that is present*) for the uncorrected cases and eventually, a compromised sensitivity on the statistical testing

Secondly, heteroskedasticity, which manifests as unequal variance, can bias the test-statistics where Type I and Type II error will increase with underestimated and overestimated standard errors respectively [19]. To further emphasize, Fig. 7a–d show the same Bowtie1, Bowtie2(global), Novoalign and BWA-mapped dilution analysis in terms of their volcano plots (i.e.*, log fold-changes* versus *p-values*). Likewise, the left and right columns show the before and after power-law correction for the 6 normalization algorithms (*arranged in row-wise*).

In each volcano plot, the noise comparisons can essentially be treated as the null hypothesis. As such, the log fold-change and p-value cutoffs (*marked by double horizontal dotted lines and single vertical dotted line*) for the purpose of deriving the significant number of transcripts in the positive comparisons, were determined from the largest absolute fold-change value and smallest p-value of these 6 noise comparisons (*in blue*). The latter aims to exclude any false-positives. Furthermore, the rate of change in p-value against fold-change can also be derived from the two cutoff values and is indicated in each volcano plot. Finally, for each of the 4 positive comparisons, the exact breakdown of the number of significant transcripts for all combinations of mapping and normalization methods before and after power-law correction were computed (see Additional file 5: Table S2 for full details).

Based on the volcano plots, the slower rate of change in p-values of the uncorrected cases when compared to the power-law corrected cases, implies that a higher fold-change threshold is required to achieve a comparable p-value (*or Type I error rate*) during statistical testing. Consequently, the higher fold-change threshold also implies a larger type II error (i.e.*, failing to detect an effect that is present*) for the uncorrected cases and hence, a compromised sensitivity on the statistical testing. Indeed, based on Table 4, the general number of significant transcripts are higher for the power-law corrected analysis than the uncorrected ones. The trend is consistent regardless of the mapping algorithms used when averaged over the 6 normalization methods for each positive comparison. Meanwhile, it should also be noted that the variation contributed by different normalization algorithms is larger than that of different mapping methods. Overall, the average increase in sensitivity (*in terms of percentage*) across the 4 comparisons after power-law correction, is between 26% to 28% (36~ 42 transcripts versus 50~ 57 transcripts) for the Bowtie1-mapped analysis, between 27% to 30% (41~ 44 transcripts versus 58~ 61 transcripts) for the Bowtie2(global)-mapped analysis, between 26% to 34% (36~ 43 transcripts versus 54~ 58 transcripts) for the Novoalign-mapped analysis and between 26% to 32% (36~ 41 transcripts versus 53~ 58 transcripts) for the BWA-mapped analysis.

**Table 4:** Median number of significant transcripts calls in the comparative dilution analysis (AGS versus NUGC3) before and after power-law correction

	Original data				Power-law corrected data			
Mapping method	AGS 12p vs NUGC3 12p	AGS 12p vs NUGC3 3p	AGS 3p vs NUGC3 12p	AGS 3p vs NUGC3 3p	AGS 12p vs NUGC3 12p	AGS 12p vs NUGC3 3p	AGS 3p vs NUGC3 12p	AGS 3p vs NUGC3 3p
Bowtie1	42	41	39	36	57	52	52	50
Bowtie2 (global)	44	43	43	41	61	59	61	58
Novoalign	43	40	39	36	58	57	57	54
BWA	41	41	39	36	58	55	56	53

The breakdown of significant transcript calls for each combination of the mapping algorithms (*Bowtie1, Bowtie2(global), Novoalign and BWA*) and normalization methods (*DESeq, RLE, TMM, Upperquartile, CPM and Quantile*) for all 4 positive comparisons (*AGS-12p* versus *NUGC-12p, AGS-12p* versus *NUGC-3p, AGS-3p* versus *NUGC-12p and AGS-3p* versus *NUGC-3p*) are given in the following table. The median number of significant calls for 6 normalization methods are highlighted in red for each mapping algorithm

### Independent validation of power-law application on the full spike-in data series

As an independent validation, the full spike-in dataset which includes the 12 non-human spike-in transcripts was also analyzed. Given 12 samples in total without technical replicates across conditions, the total number of possible pairwise comparisons is 66 cases (${C}_2^{12}$) where the positive set is made up of the 12 spike-in transcripts (or signal) while the negative set (or noise) is composed of 460 UHR transcripts after filtering for non-zero count values among the conditions. In addition, given that the original authors’ BWA-mapped counts were used, the permutation step across the 4 mapping algorithms was excluded. Also, due to the cyclic latin-square design of the spike-in transcripts across the 12 samples, the uniqueness of each sample meant that there are no replicates and hence, statistical evaluation is not possible. Instead, the cutoff criteria for significant call is simply based on the fold-change. As an additional note, the optimum PPS setting for the power-law corrected data was evaluated to be 10 according to the optimization plot (*See* Additional file 6: Figure S5B). Note that due to the lack of replicates for the spike-in transcripts, only the background set was used for the parameter estimation.

**Fig. 8:**
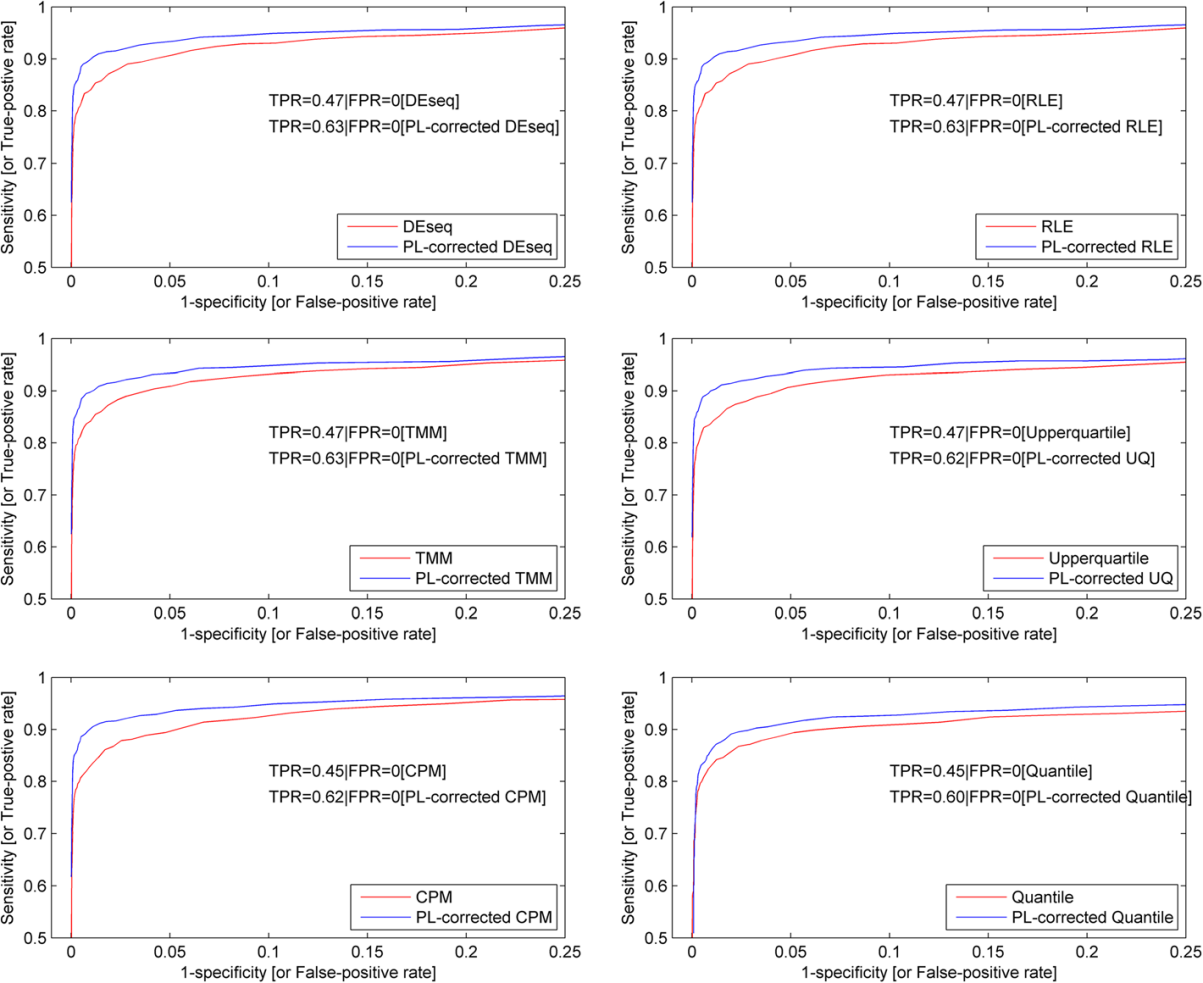
Receivers Operator Characteristics (ROC) curves of the spike-in data set before and after power-law correction. Figure 8 shows the receivers operator characteristics (ROC) curves for the 6 normalization methods: DESeq, Relative Log Expression (RLE), Trimmed Mean of M-values (TMM), UpperQuartile (UQ), Count Per Million (CPM) and Quantile normalization. For each ROC plot, the sensitivity and specificity values were derived through the permutation of the log fold-change range of the noise comparisons. The plot without correction is shown in red while the power-law corrected one is depicted in blue. Overall, an obvious improvement in the performance upon the power-law correction can be seen regardless of normalization methods. Among the methods, the quantile normalization method gave the worst performance

Figure 8 shows the receivers operator characteristics (ROC) curves for the 6 normalization methods: DESeq, Relative Log Expression (RLE), Trimmed Mean of M-values (TMM), UpperQuartile (UQ), Count Per Million (CPM) and Quantile normalization. For each ROC plot, the sensitivity and specificity values were derived through the permutation of the log fold-change range of the noise comparisons. The plot without correction is shown in red while the power-law corrected one is depicted in blue. From the ROC plots, there is an obvious improvement in the performance across all tested normalization methods after the power-law correction. Among the methods, the performance is almost comparable to one another with the exception of the quantile normalization method. Furthermore, to compare against the BWA performance of the dilution analysis, the sensitivity of the spike-in analysis for each normalization method was evaluated at the false-positive rate of 0 (*See the sensitivity values before and after power-law correction in the ROC plots*). As compared to the improvement in statistical sensitivity of 26% to 32% in the dilution analysis, the improvement in detection sensitivity for the spike-in analysis is lower (i.e.*, between 15% to 17%*) across all the methods since its undersampling condition was less severe than that of the dilution data set.

### Power-law correction improves the concordance in significant transcript call among normalization algorithms, especially with increased sequencing depth

Another important implication of the power-law correction is that the improved concordance in significant transcript call among the different normalization methods [12–14] will decrease the workflow’s dependency on the variations in specific algorithms. Returning to the dilution data set analysis, Table 5 gives the average concordance in significant calls by various mapping/normalization methods (*see* Additional file 5: Table S2 for the detail breakdown). It summarizes the level of agreement between the 6 normalization algorithms per mapping method for the positive comparisons in NGS workflow as shown in Fig. 5. Briefly, the “intersect” row gives the total number of common significant transcripts with the same fold-change directionality among the 6 algorithms, the “union” row gives the total number of significant transcripts reported by any of the 6 algorithms while the concordance ratio (*in %*) is taken between the “intersect” total and the “union” total. The concordance ratio serves as an unbiased measure given its double-edged sword nature; While an increase in significant call by all algorithms is necessary to increase the “intersect” count, it also increases the likelihood that only some of the algorithms are making the call, thus lowering the concordance ratio.

**Table 5:**
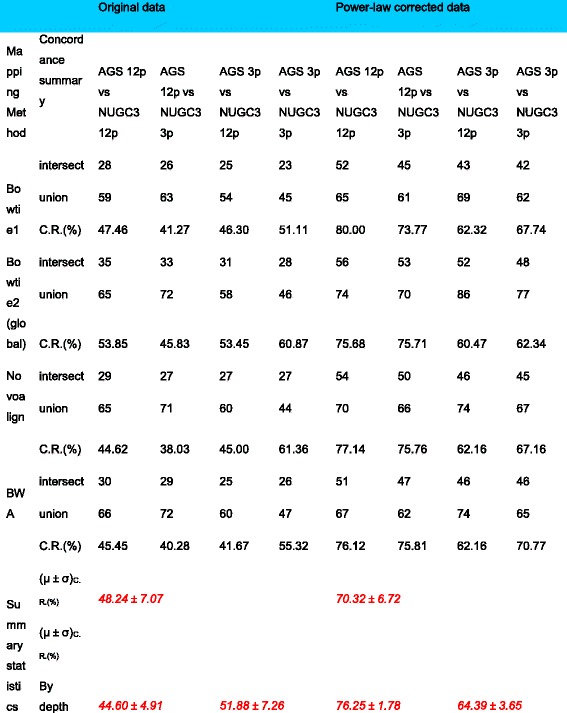
Concordance summary of significant transcripts calls of comparative dilution analysis (AGS versus NUGC3) before and after power-law correction

The following table gives the agreement of significant transcript calls among the 6 normalization methods (*DESeq, RLE, TMM, Upperquartile, CPM and Quantile*) for each mapping algorithms (*Bowtie1, Bowtie2(global), Novoalign and BWA*) for the following 4 positive comparisons: AGS-12p versus NUGC-12p, AGS-12p versus NUGC-3p, AGS-3p versus NUGC-12p and AGS-3p versus NUGC-3p. The summary statistics row gives the concordance of comparisons (i) across all sequencing depth (top row) and (ii) stratified by sequencing depth (bottom row)

With the power-law correction, the increase in the “intersect” total has almost doubled for all mapping/normalization combinations across all comparisons (*see “intersect” rows*). Meanwhile, the corresponding increase in the “union” total is less than one-quarter at its worst (see *“union” rows*). This gives an increase of about 22% in concordance rate after the power-law correction i.e., (70.32 ± 6.72)% versus (48.24 ± 7.07)% (*See “summary statistics” first row in* Table 5). When the comparisons are further stratified by their sequencing depths (i.e.*, AGS-12p and AGS-3p comparisons*), an increase in sequencing depth does not necessarily improve the concordance rates. In fact, the higher sequencing depth AGS-12p instance has a lower concordance rate of (44.6 ± 4.91)% than that of the lower sequencing instance at (51.88 ± 7.26)% (*See “summary statistics” second row in* Table 5). In retrospect, although the number of significant transcript calls or the “intersect” total has generally increased with a higher sequencing depth, the inconsistency in significant transcript calls among the various normalization methods (i.e.*, the “union” total*) has increased at a faster rate which resulted in a lower concordance rate despite the higher sequencing depth.

With the power-law correction, a higher sequencing depth correctly returns a higher concordance rate. Between the uncorrected and power-law corrected analysis, the improvement is somewhat asymmetrical where it was about 32% (44.6% ± 4.91% versus 76.25% ± 1.78%) for the higher sequencing depth AGS-12p instance while this was about 13% (51.88% ± 7.26% versus 64.39% ± 3.65%) for the lower depth AGS-3p instance. It remains that sufficient sequencing depth is necessary to generate enough information but when the condition is met, power-law correction will be able to extract any additional information content to increase significant detection.

### Enhanced statistical conclusions elucidates the metastatic potential of the NUGC3 gastric cancer cell line

While both AGS and NUGC3 cell lines were commonly described as gastric adenocarcinoma according to the Cellosaurus database (*version 22;* *http://web.expasy.org/cellosaurus/*), NUGC3 was derived from a distal metastasis site - the Brachialis muscle of a male patient and AGS is presumably taken from the primary site of a female patient. Therefore, their comparison should elude the metastasis potential of the NUGC3 cell line beyond the common gastric adenocarcinoma. According to current literature, the common metastasis site of stomach cancer (in ascending order) is the liver, peritoneum, lung and bone [34, 35] while it is considerably rare to spread to the pancreas and skeletal muscle [36, 37]. When compared to generic adenocarcinoma which often spreads to the liver and lung [38], signet-ring adenocarcinoma frequently metastasizes within the peritoneum, bone, ovaries and sometimes to the breast [34, 39].

In our comparative study of the two gastric cell lines, the Bowtie1-mapped concordance transcripts from Table 5 before and after power-law correction were independently subjected to gene-set enrichment analysis (GSEA) via the MiEAA webserver to identify plausible disease groups from the collection of Human microRNA and Disease Database (HMDD). Briefly, using the Bowtie1-mapped results from Table 5, the concordance transcripts across the 4 comparisons before power-law correction (*see “intersect row”; columns 3–6*) were compiled into a union set of concordance transcripts. The same was done for the power-law corrected comparisons (*see “intersect row”; columns 7–10*). Altogether, the uncorrected and power-law corrected union sets consist of 30 and 52 concordance pre-cursor miRNA transcripts respectively (*see* Additional file 7: Table S3 columns 1 and 2). The uncorrected list exceeded the maximum intersect value of 28 (*AGS-12p* versus *NUGC3–12p*) due to some slight variations among the 4 comparisons. Between the two concordance sets, the uncorrected set is almost a complete subset of the corrected set; one transcript is unique to the uncorrected set while this was 23 for the corrected set (*See* Additional file 7: Table S3 columns 3 and 4).

Thereafter, both lists were independently subjected to gene-set enrichment analysis (GSEA) via the MiEAA webserver to identify plausible disease groups from the collection of Human microRNA and Disease Database (HMDD). For the power-law corrected list, the specific parameters are as follows: count≥10 and FDR-adjusted *p* ≤ 0.05; This gives a maximum expected value of 0.5 for false-positives (FP). To match the FP count of 0.5, the necessary parameters for the uncorrected list are: count≥5 and FDR-adjusted *p* ≤ 0.1 (*See* Table 6 *legend for detailed explanation*).

**Table 6:**
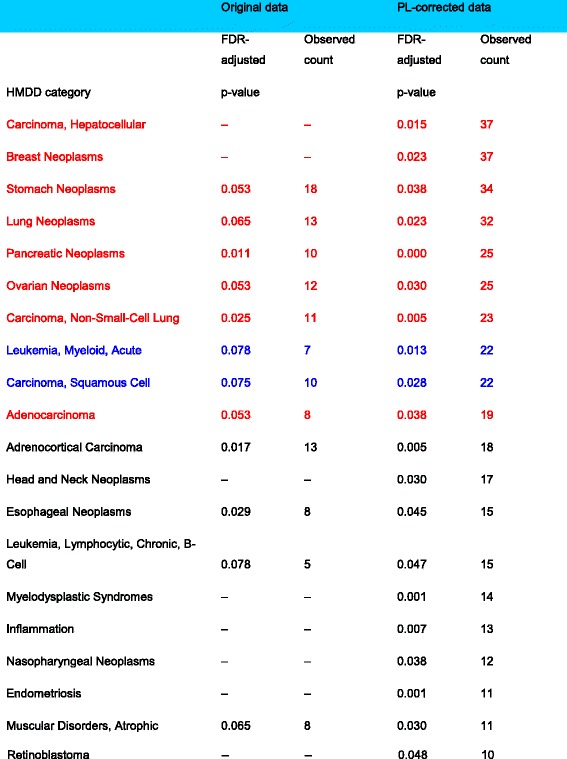
miRNA enrichment of concordance transcripts before and after power-law correction

This table gives the gene-set enrichment analysis (GSEA) in the significant HMDD (Human microRNA and Disease Database) categories of the Bowtie1-mapped uncorrected and power-law corrected concordance transcripts (total of 30 and 52 respectively) as listed in Table 5, via the MiEAA webserver. For the power-law corrected list, the specific parameters are as follows: count≥10 and FDR-adjusted *p* ≤ 0.05; This gives a maximum expected value of 0.5 for false-positives (FP). To match the FP count of 0.5, the necessary parameters for the uncorrected list are: count≥5 (approximated from 10/52*30 = 5.77 where 10/52 is the ratio of power-corrected count of 10 over its total concordance transcripts of 52) and FDR-adjusted *p* ≤ 0.1 (approximated from 0.5/5.77 = 0.08). The identified HMDD categories from the two MiEAA runs were sorted by observed count, then FDR-adjusted p-value based on the power-law corrected results. Categories highlighted in red, blue and black are denoted as significant, significant false-positives and non-significant

Table 6 consolidates the identified HMDD categories of both analysis sorted by observed count, then by FDR-adjusted *p*-value. The expected baseline category - “adenocarcinoma” was used as the cutoff point for significance and hence, any categories beyond it were considered as insignificant hits (*marked in black*). Within the significant categories, there are two likely false-positive hits (*marked in blue*). They are the “Leukemia, Myeloid, Acute” hit that should be grouped with the non-significant “Leukemia, Lymphocytic, Chronic” and the “Carcinoma, Squamous Cell” hit that should group with the non-significant “Esophageal Neoplasms” hit to explain esophageal cancer.

Between the uncorrected and power-law corrected result sets, the latter presents the stronger evidence of expected gastric adenocarcinoma through its more significant *p*-values for both “stomach neoplasms” and “adenocarcinoma”. Likewise, the remaining significant hits suggest several neoplasms and carcinoma (*“lung neoplasms”, “pancreatic neoplasm”, “ovarian neoplasm” “carcinoma, non-small-cell lung”*) as possible metastasis sites for NUGC3 with stronger statistical conclusions being drawn from power-law corrected analysis. In addition, power-law analysis discovers two more metastasis categories - “carcinoma, hepatocellular” and “breast neoplasms” with significant *p*-values 0.015 and 0.023 respectively. Overall, the power-law corrected analysis concurs significantly better with the clinical evidence.

## Conclusion

Specifically, our work has identified and mathematically quantified an important technical limitation of the sequencing technology for transcriptomics applications where finite-size effects due to undersampling [15, 29] can have profound effects on the reproducibility and statistical qualities of underlying transcript abundance distribution for its subsequent interpretation; This is independent of the advancement in sequencing technology since sampling is finite in the real world. With a simple distribution correction, the signal-to-noise ratio and sensitivity of statistical detection in a typical comparative analysis can experience an instant and dramatic improvement that greatly impacts the reliability of the final biological interpretation of the study.

## Methods

### Property of type I Pareto distribution

When transcript abundance is being visualized in a rank-frequency plot, the Zip’s law [3–7] is specifically being singled out. Meanwhile, there exists a close relationship between the family of Pareto distributions (*Type I, II, II and IV*) to the Zip’s law; Type II to IV Pareto distributions varied from Type I mainly from the addition of a location and shape parameter that are irrelevant to the modelling of transcript abundance. Among the Pareto family, the Type I Pareto distribution remains the most mathematically compatible to the rank-frequency plot where their two axis can be shown to be interchangeable (*See methods for details*: *Transformation between rank-frequency and Pareto distribution*).

Mathematically, the probability (PDF) and cumulative (CDF) density function of the Type I Pareto distribution are defined as:

1 $$ P\left(X=x;{x}_{\mathrm{min}},s\right)=\frac{sx_{\mathrm{min}}^s}{x^{s+1}} $$

2 $$ P\left(X\le x;{x}_{\mathrm{min}},s\right)=\left\{\begin{array}{ccc}1-{\left(\frac{x_{\mathrm{min}}}{x}\right)}^s& for& x\ge {x}_{\mathrm{min}}\\ {}& & \\ {}0& for& x<{x}_{\mathrm{min}}\end{array}\right. $$

for the interval *x* ≥ *x*_min_ and *x*_min_ is the minimum value of the distribution and is necessarily positive (i.e. *x*_min_ > 0). In addition, the Pareto’s tail distribution (*complementary CDF*) is simply defined as *P*(*X* > *x*). Correspondingly, the mean and variance of the Pareto distribution are given as:

3 $$ \mu =\left\{\begin{array}{ccc}\frac{sx_{\mathrm{min}}}{s-1}& for& s>1\\ {}& & \\ {}\infty & for& s\le 1\end{array}\right. $$

4 $$ {\sigma}^2=\left\{\begin{array}{ccc}\frac{sx_{\mathrm{min}}^2}{{\left(s-1\right)}^2\left(s-2\right)}& for& s>2\\ {}& & \\ {}\infty & for& 0<s\le 2\end{array}\right. $$

Therefore, for large values of the exponent term *s*, the corresponding mean *μ* and variance term *σ*^2^ converges towards smaller values for a fixed *x*_min_.

### Derivation of the alias term in the power-law 1/*f*^*α*^ equation

Aliasing refers to a distortion or an artifact when a reconstructed signal differs from its original continuous signal. In this section, the alias term for the power-law equation 1/*f*^*α*^ is derived. Note that the main derivation originates from Kirchner [29] and this section provides only a concise adaptation.

Given a time series *x*(*t*), its Fourier transform of its discrete sampled time series *y*(*t*) is given as:

5 $$ Y(f)=\underset{-\infty }{\overset{\infty }{\int }}x(t) III(t){e}^{-i2\Pi ft} dt $$

Furthermore, given that the sampling function *III*(*t*) is a periodic function at a sampling interval of Δ*t* = 1/*f*_*s*_, it can be defined as:

6 $$ III(t)=\sum \limits_{-\infty}^{\infty }{c}_k{e}^{i2\Pi {kf}_st} $$

where ${c}_k=\frac{1}{\Delta t}\underset{-\Delta t/2}{\overset{\Delta t/2}{\int }}\partial \left({f}_st\right){e}^{-i2\Pi {kf}_st} dt=\frac{1}{\Delta t}\frac{1}{f_s}=1$ for all k.

Combining Eqs. (5) and (6), one can re-express the Fourier transform of *y*(*t*) into:

7 $$ Y(f)=\underset{-\infty }{\overset{\infty }{\int }}\sum \limits_{k=-\infty}^{\infty }{e}^{i2\Pi {kf}_st}x(t){e}^{-i2\Pi {f}_st} dt=\underset{-\infty }{\overset{\infty }{\int }}\sum \limits_{k=-\infty}^{\infty }x(t){e}^{-i2\Pi \left(f-{kf}_s\right)t} dt $$

Also, given that the summation is taken over all k, the term −*kf*_*s*_ can replace by *kf*_*s*_. Together with interchanging the summation and integration sign, one yields the following:

8 $$ Y(f)=\sum \limits_{k=-\infty}^{\infty}\underset{-\infty }{\overset{\infty }{\int }}x(t){e}^{-i2\Pi \left(f+{kf}_s\right)t} dt=\sum \limits_{k=-\infty}^{\infty }X\left(f+{kf}_s\right) $$

In addition, the sampled function *Y*(*f*) can be decomposed into its original signal *X*(*f*) and its alias components as follows:

9 $$ Y(f)=X(f)+\sum \limits_{k=-\infty, k\ne 0}^{\infty }X\left(f+{kf}_s\right) $$

Since *x*(*t*) is a real function, its Fourier transform *X*(*f*) is Hermitian. Therefore, *X*(−*f*) = *X*(*f*) and Eq. (9) can be written for positive frequencies only as follows:

10 $$ Y(f)=X(f)+\sum \limits_{k=1}^{\infty }X\left({kf}_s-f\right)+X\left({kf}_s+f\right) $$

Substituting the power-law equation *X*(*f*) = *S*_*o*_*f*^−*α*^ into (10) yields:

11 $$ Y(f)={S}_o{f}^{-\alpha }+\sum \limits_{k=1}^{\infty }{S}_o{\left({kf}_s-f\right)}^{-\alpha }+\sum \limits_{k=1}^{\infty }{S}_o{\left({kf}_s+f\right)}^{-\alpha } $$

For Eq. (11) to converge mathematically, (i) the high frequency component (*kf*_*s*_ + *f*) cannot be extended infinitely; In real-world, high frequency components fall off faster than 1/*f*^*α*^ way above the sampling frequency) and (ii) the condition where *α* > 1 needs to be satisfied. Hence, the Fourier transform of *x*(*t*) can be simplified to the following form:

12 $$ Y(f)={S}_o{f}^{-\alpha }+\sum \limits_{k=1}^{\infty }{S}_o{\left({kf}_s-f\right)}^{-\alpha } $$

Furthermore, for a band-limited signal of 0 ≤ *f* ≤ *f*_max_, the only relevant alias term is (*f*_*s*_ − *f*_max_) where *k* = 1, since (*kf*_*s*_ − *f*_max_) > 0 will satisfy the Nyquist sampling criterion of *f*_max_ < *kf*_*s*_ for which *k* ≥ 2. In other words, aliasing will not occur for *k* ≥ 2. Finally, the power-law Fourier series of *x*(*t*) with the relevant alias term when undersampling occurs, is given as:

13 $$ Y(f)={S}_o{f}^{-\alpha }+{S}_o{\left({f}_s-f\right)}^{-\alpha } $$

where *Y*(*f*) is the sampled function, *S*_*o*_*f*^−*α*^ is the original signal and *S*_*o*_(*f*_*s*_ − *f*)^−*α*^ is the alias component.

### Transformation between rank-frequency and Pareto (type I) distribution

The Pareto (Type I to IV) distribution belongs to the large family of power-law distributions; the subsequent derivation refers specifically to the Type I Pareto distribution. Given an observation, the Pareto’s tail distribution (*complementary CDF*) describes how many cases are seen greater than the observation in terms of cumulative density function (CDF). Meanwhile, the rank-frequency distribution is an inverse CDF (*quantile function*) seen in a reverse order with respect to the Pareto distribution, where it depicts the occurrence of the observation at a given rank.

First, let the rank-frequency equation be defined as:

14 $$ x={C}_1{y}^{-b} $$

where *y* is a *y*^*th*^ ranked value and *x* is the number of observed occurrences at *y*. One can further implies that there exists *y* number of values for which their corresponding *x* values are greater than *C*_1_*y*^−*b*^. As such, one can write a cumulative density function for random variable *X* for the number of observations larger than *C*_1_*y*^−*b*^ in the form:

15 $$ P\left(X>{C}_1{y}^{-b}\right)={C}_2y $$

where *C*_2_ is a normalization constant such that *P*(*X* ≥ *C*_1_*y*^−*b*^) ≤ 1 must be satisfied. Then, rearranging Eq. (14) into $y={\left[\frac{x}{C_1}\right]}^{-\frac{1}{b}}$ and substituting it into Eq. (15) yields the Pareto’s tail distribution or complementary CDF:

16 $$ P\left(X>x\right)={C}_2{\left[\frac{x}{C_1}\right]}^{-\frac{1}{b}} $$

For completeness sake, one can replace ${x}_{\mathrm{min}}={C}_1{C}_2^b$ to obtain the usual Pareto’s tail distribution form of $P\left(X>x\right)={\left[\frac{x}{x_{\mathrm{min}}}\right]}^{-\frac{1}{b}}$ for *x* ≥ *x*_min_. Meanwhile, to convert from the complementary CDF to the complementary cumulative total function (CTF), the expression can simply be rearranged as follows:

17 $$ y=\frac{1}{C_2}\cdot P\left(X>x\right)={C}_1^{-\frac{1}{b}}{x}^{-\frac{1}{b}} $$

Hence, comparing terms in Eqs. (14) and (17), it can be seen that the Pareto’s tail distribution (in terms of complementary CTF) and rank-frequency distribution are inversely related.

### Solving for sampling frequency *f*_*s*_ to determine undersampling

Taking logarithm on both sides of Eq. (13), the sampled function *Y*(*f*) can be rewritten in logarithmic form as:

18 $$ \log Y(f)=\log \left[{S}_o{f}^{-\alpha}\right]+\log \left[\frac{S_o{f}^{-\alpha }+{S}_o{\left({f}_s-f\right)}^{-\alpha }}{S_o{f}^{-\alpha }}\right] $$

The second term on the right hand-side gives a distortion ratio between an aliased signal *S*_*o*_*f*^−*α*^ + *S*_*o*_(*f*_*s*_ − *f*)^−*α*^ and original signal *S*_*o*_*f*^−*α*^. As such, let the distortion ratio Δ*Y*(*f*) be defined as:

19 $$ \Delta Y(f)=\frac{S_o{f}^{-\alpha }+{S}_o{\left({f}_s-f\right)}^{-\alpha }}{S_o{f}^{-\alpha }} $$

Further simplification yields:

20 $$ \Delta Y(f)=1+\frac{{\left({f}_s-f\right)}^{-\alpha }}{f^{-\alpha }} $$

And solving for the sampling frequency *f*_*s*_ gives:

21 $$ {f}_s=f+f\cdot {\left[\Delta Y(f)-1\right]}^{-\frac{1}{\alpha }} $$

For a rank-frequency plot where Zipf’s law holds (i.e.*, α* = 1), *f*_*s*_ can directly be evaluated when *f* = *f*_max_, Δ*Y*(*f*) = Δ*Y*(*f*_max_).

### Derivation of the power-law correction factor

In an earlier section, the rank-frequency distribution and Pareto’s tail distribution has been proven to be inversely related to each other. For the purpose of estimating the exponent term in the rank-frequency plot, a better approach is to use Pareto’s tail distribution. This is because the large-ranked tail of rank-frequency distribution tend to be clustered with small values of the same rank. As a result, this give a horizontal tail. In contrast, the same segment is always monotonically-increasing in Pareto. As such, let the count and rank of the *i*^*th*^ transcript be x and y respectively. Then the rank-frequency equation in its Pareto’s tail distribution form or complementary CTF can be written as.

22 $$ y={kx}^{-s} $$

where *y* = *C*_2_ ⋅ *P*(*X* ≥ *x*), $k={C}_1^{-s}$ and $s=\frac{1}{b}$ from Eq. (17).

Taking logarithm on both sides, the expression is rewritten as:

23 $$ {\log}_by={\log}_bk+m{\log}_bx $$

where the slope and intercept are represented by *m* =  − *s* and log_*b*_*k* respectively. Then, to convert the original slope and intercept (*m*, log_*b*_*k*) to a reference set of parameters (*m*_*ref*_, log_*b*_*k*_*ref*_), we let:

24 $$ {\displaystyle \begin{array}{l}{\log}_by=\left({\log}_bk-{\log}_b{k}_{ref}\right)+{\log}_b{k}_{ref}+{m}_{ref}\left(\frac{m}{m_{ref}}\right){\log}_bx\\ {}{\log}_by={\log}_b{k}_{ref}+{\log}_b\left(\frac{k}{k_{ref}}{x}^{m_{ref}\left(\frac{m}{m_{ref}}\right)}\right)\end{array}} $$

In the original scale, the rank-frequency equation can be re-expressed as:

25 $$ y={k}_{ref}{\left[{\left(\frac{k}{k_{ref}}\right)}^{\frac{1}{m_{ref}}}{x}^{\frac{m}{m_{ref}}}\right]}^{m_{ref}} $$

Finally, the corrected count *x*^'^ is given as:

26 $$ {x}^{\hbox{'}}={\left(\frac{k}{k_{ref}}\right)}^{\frac{1}{m_{ref}}}{x}^{\frac{m}{m_{ref}}} $$

The power-law correction is implemented in PERL language and can be downloaded from the supplementary website [22].

### Computation procedures for power-law correction of a count data set

The restoration of an observed distribution towards an uniform power-law entails that the slopes of all count segments to be the same. The reference power-law slope is taken from the highest-count segment since this segment is sampled from the higher abundance transcripts and should have the best mathematical convergence towards its real value. And with the correction towards a common slope, it is expected that all count segments will have similar variation among the replicates and that the overall heteroskedasticity should be dramatically reduced. Without the loss of generality, the proposed sequencing count correction will be, herein, named as the power-law correction.

In the actual implementation of the power-law correction procedure, there are two important computational aspects to note. Firstly, for the purpose of estimating the exponent term in a rank-frequency plot, the Pareto equation (*see Eq. 21*) is used rather than Zipf’s (*see Eq. 14*) because the large-ranked tail of Zipf’s law tends to be clustered with small values of the same rank. As a result, this gives a horizontal tail which is sub-optimal for slope estimation. In contrast, the same segment is always monotonically increasing in Pareto.

Secondly, the power-law correction is performed at a per-sample level. The total number of count segments in a Pareto plot is dependent on a fixed number of points per segment, herein, as points-per-segment (PPS). The partitioning of points will start from the highest count value. For each partitioned count segment, a set of slope and intercept (*m*, log_*b*_*k*) values will be solved using linear regression (see Eq. 22). The first-fitted count segment of the replicate which mimics the highest-count segment, will be used as the reference set of slope and intercept (*m*_*ref*_, log_*b*_*k*_*ref*_) values for the subsequent power-law correction via Eq. 26.

To find the optimum PPS setting that will yield the best overall fit between any replicate to a reference replicate in a N-sample dataset, the PPS parameter first needs to be permuted across a range of between 5 to 100 at an interval of 5. At a given PPS setting, two measures can be derived. First, the median of the N first-fitted count segment slopes of the data series can be taken. Secondly, a total of (N-1) R^2^ (i.e.*, coefficient of determination*) values can be derived from the linear regression results between the N-1 replicates against the reference replicate. Consequently, a median R^2^ can also be taken.

The preceding computational procedures were then applied to the original BWA-mapped spike-in background and Bowtie1-mapped NUGC3 dilution data. Additional file 8*:* Figure S4A and S4B show the median slope of the first-fitted segments versus the median R^2^ value of the spike-in background set and the NUGC3 dilution set respectively. The PPS values are indicated besides the data points in the plots. Like before, the reference replicate was taken as the replicate with the largest total reads within the data series for the necessary R^2^ computations. For both Figures, the refined solution space of the optimum PPS is indicated by the error margins defined by the slope of the first highest-count segments from Table 1. Within this margin, the optimum PPS value is determined by the largest median R^2^ value. As such, the optimum PPS settings for the spike-in background set and the NUGC3 dilution set are 20 and 45 respectively. The subsequent analysis is then based on the power-law corrected data sets using these PPS settings and their associated median slopes as the reference slope values for the respective data series. Similarly, the procedures were also applied to the BWA-mapped spike-in and Bowtie1-mapped full dilution data sets to obtain the optimum parameters (*see* Additional file 6: Figure S5A and S5B). The parameter sets were subsequently used on the Bowtie2(global)-mapped, Novoalign-mapped and BWA-mapped full dilution data sets to generate the results in Table 3.

### The dilution dataset

Overview of design: The dilution series was created for two gastric cancer cell lines - AGS and NUGC3. The NUGC3 set consists of 8 replicates and spans across 4 concentration points of 12p, 6p, 3p and 1.5p so that each concentration contains exactly two technical replicates. Meanwhile, the AGS set is similarly designed except that it consists of 4 replicates across 2 concentrations of 12p and 3p. The varying concentration design aims to simulate the different sequencing depth (i.e.*, the total mapped reads*) that mimics a system of various sizes to study its finite-size effects. The original sequencing files (*in FASTQ format*) of this dilution dataset can be downloaded from the supplementary website [22].

Sample preparation (Total RNA extraction): Isolation of total RNA from AGS and NUGC3 was performed using a Qiagen miRNeasy mini kit (Qiagen). Briefly, 5× volume of QIAzol lysis reagent was added to 1 million cells, incubated at room temperature for 5 min to disrupt and homogenize the cells. 1 volume of chloroform is then added to the tube, shaking vigorously for 15 s and incubates at room temperature for 2–3 min. Mixture is then transferred to a 2 ml Qiagen MaXtract high density tube and centrifuged for 15 min at 12,000 g for phase separation. Upper aqueous phase is carefully transferred to a new collection tube and 1.5 volume of 100% ethanol is added to aqueous phase for precipitation of total RNA in aqueous phase. The mixture is then pass into the RNeasy mini elute spin column (700ul each time) placed in a 2 ml collection tube. The column is spin at ≥8000 g for 15 s at room temperature and flow through is discarded. Process is repeated until all mixture has pass through column. Column is washed with 700ul of Buffer RWT and centrifuged at ≥8000 g for 15 s at room temperature Column is further washed with 500ul of Buffer RPE, spin at ≥8000 g for 15 s at room temperature. Lastly, column is washed with 500ul of 100% ethanol, centrifuge for 2 min at ≥8000 g. Column is transferred to a new collection tube and spin at ≥8000 g for 5 min at room temperature to remove residual ethanol and total RNA elute in 10ul of RNase-free water.

TruSeq small RNA library construction and sequencing: 6 (4 for NUGC3 and 2 for AGS) small RNA libraries were prepared in parallel for both NUGC3 and AGS cell lines using the Illumina TruSeq small RNA sample preparation kit according to manufacturer’s instruction. The 6 samples were uniquely indexed to enable sequencing of all 6 libraries in one MiSeq flow cell. Briefly, 1μg of total RNA was ligated with 5′ and 3′ adapter, cDNA was converted with SuperScript II Reverse Transcriptase and RT Primer. The cDNA was PCR amplified for 12 cycles with RNA PCR Primer and unique PCR Primer Index provided; It is important to note that indexing during PCR amplification minimizes the issue of barcoding bias [40] which masks significant expression differences between miRNA libraries. Amplified cDNA construct were first purified using QIagen MinElute PCR Purification kit and the construct were then size selected for fragments ranging between 145 bp to 150 bp using 10% TBE PAGE Gel. The indexed libraries were quantified individually by qPCR using KAPA SYBR FAST qPCR Kit (Kapa Biosciences, inc). To stimulate differences in sequencing depth in a multiplex sequencing experiment, the small RNA libraries for the NUGC3 cell line were pooled such that there was a 1, 2, 4 and 8× difference in concentration between the four unique libraries (12pM, 6pM, 3pM, 1.5pM). Small RNA libraries for AGS was pooled such that there is a 4× difference in concentration between the two unique libraries (12pM and 3pM). The libraries from both cell lines were pooled to yield a single pooled library and sequenced twice on the MiSeq instrument using MiSeq Reagent v2 for 1 × 40 + 6 (index) sequencing cycle (Illumina Inc., CA, USA).

### Generalized NGS comparative workflow

Read mapping:

Raw data in FASTQ format was preprocessed using Trimmomatic [41] version 0.33 by trimming adapter sequences, removing trailing or leading low quality bases (base quality below 3). Subsequently, scan the reads with a 4-base wide sliding window and trim when the average base quality drops below 15. Specifically, the command for Trimmomatic is:

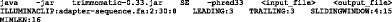



The preprocessed reads were then aligned to miRBase v21 primary sequences using three different aligners, i.e. Bowtie (version 1.1.1 and 2.3.0) [30], Novoalign (*www.novocraft.com*; version V3.04.06) and BWA (version 0.7.12-r1039) [31, 32] with the specific parameters as shown below:

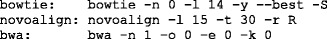



Aligned reads in BAM format is then quantified using BEDtools [42] by counting how many reads map to each of the miRNA transcript. The respective mapped count files can be downloaded from the supplementary website [22].

For normalization, the EdgeR, DESeq and preprocessCore R packages were used in this work. Prior to normalization, the data is first organized into its specific cell lines (NUGC3, AGS) and concentration (12pM, 6pM, 3pM, 1.5pM) groups of 2 technical replicates via the following command:

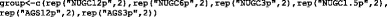



Next, the data is read from an input file to perform the specific normalization. At the same time, an EdgeR DGElist object and the associated normalization factors for the proper scaling of the raw library sizes will also be created.

For DESeq normalization, the combined commands are as follows:

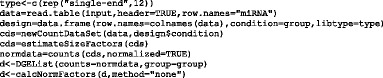



For Quantile normalization, the combined commands are as follows:

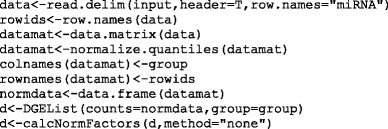



For CPM normalization, the combined commands are as follows:

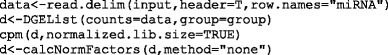



For TMM, RLE, upperquartile normalization where m takes one of the following values “TMM”,“RLE”,“upperquartile”, the commands are as follows:

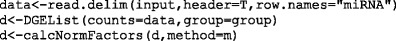



For performing statistical analysis, the generalized linear model (GLM) [33] from the EdgeR package was used. First, the count data is first fitted to the negative binomial model in the EdgeR package [26] for the purpose of estimating the common and tag dispersion. This is achieved through the Cox-Reid profile-adjusted likelihood methods via the following commands:

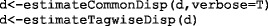



Next, to allow for multiple contrasts in the comparison of AGS cell line against NUGC3 cell line, the GLM design matrix is to be set up. Specific to the dilution data set, the 4 treatment contrasts are AGS-12p versus NUGC-12p, AGS-12p versus NUGC-3p, AGS-3p versus NUGC-12p and AGS-3p versus NUGC-3p while the 6 control contrasts are NUGC3-6p versus NUGC3–12p, NUGC3–3 versus NUGC3–12p, NUGC3–1.5p versus NUGC3–12p, NUGC3–3p versus NUGC3-6p, NUGC3–1.5p versus NUGC3-6p and NUGC3–1.5p versus NUGC3–3p. This translates to the following commands:

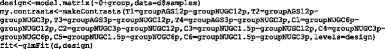



To perform the GLM likelihood test for the 4 treatments and 6 controls, the following commands were issued:

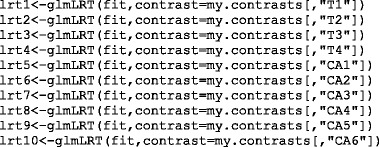



## Reviewers’ comments

### Reviewer’s report 1: Oliviero Carugo, University of Vienna, Austria

The manuscript submitted by Wong and coworkers describes a computational technique that minimizes finite-size effects in NGS datasets and robustly improves the reproducibility of the results. It is an interesting example of how statistical tools may distort reality (see for example an article on Nature today: https://www.nature.com/articles/d41586-017-07522-z) and should be used with extreme caution. It is also a nice example of how statistics begins when science ends. The methodology is described with high accuracy as well as the tests performed with both in-house and publicly available NGS data. Although very long and perhaps prolix and although the math level is probably inaccessible to most of the Biology Direct readers, I think that this manuscript deserves publication because it might inspire further research in this field.

Authors’ response: *We thank the reviewer for his positive comments. The concept behind the observed power-law distortion required a rigorous treatment as it has never been addressed in current literature and therefore, the length of the article. At the same time, we agree that the mathematics seems complex yet it was necessary for a complete treatment of the topic. Interestingly, even specialized bioinformatics journals shy away from our findings due to its lack of perceived appeal to readers attributed by the heavy mathematical contents; Regrettably, the mathematics cannot be further simplified. Taken together, we deeply appreciate the reviewer for his support of this manuscript.*

### Reviewer’s report 2: Thomas Dandekar, Department of Bioinformatics, University of Wuerzburg, Germany

I have the following comments: At present I would think the normal reader (non mathematician) realizes: "yes, this could be an important correction, but I am not sure.".

1) So I think everything which makes the article easier to understand and more accessible would be nice. First of all, explain Zipf’s law. It is a power law probability distribution. Thus the frequency of any word is inversely proportional to its rank in the frequency table (at least like this the linguist Zipf stumbled upon it). Thus the most frequent word will occur approximately twice as often as the second most frequent word, three times as often as the third most frequent word, etc.: the rank-frequency distribution is an inverse relation.

Authors’ response: *We have expanded the Zipf’s law explanation in the first paragraph of the “Background” section to give the readers a better understanding of the origin and characteristics of Zipf’s law.*

2) I recommend I would start the article results section with a figure explaining and showing the assumed Zipf distribution regarding the sequence count data and then illustrate in the same figure how now the corrected distribution looks like (the property of type I Pareto distribution). Furthermore, it is critical to show now how the observed distribution of tag counts for the sequencing data set looks like. Ideally for the reader then it should be readily to grasp that the new function really fits better the observed data and this message should be transported by the introductory figure of the results.

Authors’ response: *Although less intuitive than the reviewer’s suggestion, we have added the Zipf’s distribution to show how the original observed distribution deviates from Zipf’s law (see dashed lines in* Fig. 1a and b*) and how the corrected observed distribution now coincides with the Zipf’s law (see dashed lines in* Fig. 3a and b*). The necessary text has also been added to the associated section where the figures were being discussed.*

*Mainly, what we wanted to achieve in the introductory message of the results section is to (i) show the observed distribution which suffers from a curvature will fit an under-sampled power-law equation Y*(*f*) = *S*_*o*_*f*^−*α*^ + *S*_*o*_(*f*_*s*_ − *f*)^−*α*^*(eq. 13) and that (ii) correcting the alias noise reverts the distribution to the form Y*(*f*) = *S*_*o*_*f*^−*α*^*(analogous to x* = *C*_1_*y*^−*b*^*(eq. 14) of the rank-frequency plot). As a consequence, the corrected observed data now fits better with Zipf’s law (*i.e.*, x* = *C*_1_*y*^−*b*^*where b ≈ 1) as shown in* Fig. 3a and b*.*

3) Another point is whether that correction is the best possible correction: could it for instance not be possible to find the best distribution by some data-driven modelling? 3b) Or are there some analytical results available why for instance a type II Pareto distribution would perform less?

Authors’ response: *The reviewer brought up an interesting issue on the interplay between the data-driven approach and model-driven (*i.e.*, analytical forms) approach. On one hand, current sequencing-based transcriptome data suffers from inherent undersampling issue which has a direct impact on the distributional shape and hence, a purely data-driven approach is not optimal. Meanwhile, a purely model-based approach to force all segments in a distribution towards a strict Zipf’s law without a good justification can be overbearing and might lead to overfitting. In our work, we balance between both data-driven and model-driven approaches by correcting the middle and tail segment of the distribution (*i.e.*, model-driven) towards the exponent value of the fitted (i.e, data-driven) high-abundance segment of the distribution which incidentally and approximately obeys the Zipf’s law.*

*As a side note, Pareto Type I has a direct 1:1 relationship to the Zipf’s law and has a support from x* ∈ [*x*_min_, ∞)*. For modelling transcript count which necessarily starts from at least one (*i.e.*, x*_min_ ≥ 1*), Pareto Type I (or Zipf’s law) seems to be the most apt distribution within the Pareto family. Meanwhile, Pareto Type II (or Lomax distribution) is simply a shifted Type I such that its support starts from 0. Mathematically, it is as follows:*

$$ P\left(X=x;{x}_{\mathrm{min}}\ge 0,s\right)=\frac{sx_{\mathrm{min}}^s}{{\left(x+{x}_{\mathrm{min}}\right)}^{s+1}} $$

*For modelling transcript count, the extra range of 0 to 1 has no relevance.*

4) The confidence of the reader would increase if you can claim that you present the current dataset but you have the correction on e.g. ten other, unrelated data sets and each time the type I Pareto distribution was the best. 4b) Even better would be to rationalize the assumed correction by the typical distribution of sequences. p.10 does something in this direction, but what I was thinking of is more a physical explanation and best taking into account specifics of the used NGS technique, for instance may be with pacific biosciences sequencing the correction should be completely different, right?

Authors’ response: *To recapitulate, SAGE-based messenger RNA data fits Type I Pareto distribution, particularly the Zip’s law relatively well* [3–7] *other than the low abundance tail segments. Independently of previous findings, we also found that NGS-based microRNA data follows the same trend in this work. When we investigated the NGS-based messenger RNA (GSE47774) of the Universal Human Reference (UHR), we found that Zipf’s law holds approximately for both the middle segments of the observed distributions (see* Additional file 9: Figure S6*) despite the differences in count quantification approach between HTSeq* [43] *and RSEM* [44] *(*i.e.*, conservative* versus *greedy mapping approach). Expectedly, the low abundance segments exhibit curvatures albeit different in their slope trends.*

*Of particular interest is that the highest and high segments in NGS-based messenger RNA data tends to exhibit steeper slopes than the Zipf’s law which characterizes the SAGE-based messenger RNA data. Preliminary conclusions suggests that this is attributed to transcript-length bias in NGS-based sequencing that is absent in SAGE-based sequencing for the messenger RNA species* [9]*. In other words, these high and highest NGS-based segments suffer from over-estimated counts that arise from abundant transcripts with multiple pair-end reads due to longer transcript lengths. As a side note, the differences in the slope trends for the low, high and highest segments between the HTSeq and RSEM quantified distributions implies that quantification algorithms generally do introduce bias in the count estimates and impacts on distributional shapes.*

*Nevertheless, regardless of the differences in technology (SAGE* versus *NGS), RNA species (microRNA* versus *messenger RNA) and count quantification algorithms (HTSeq* versus *RSEM), there exists common segments in the distribution that seems to follow the Zip’s law (*i.e.*, a specific instance of Type I Pareto distribution where its exponent term equals to 1) in our preliminary investigations. However, a generalization of Zipf’s law on transcript distribution over all types of conditions will require a separate and more thorough investigation that is beyond the scope of this manuscript.*

5) Apart from the questions I raise here I personally am convinced that such a correction is important and basically does the right thing. So another good point to spread the word would be to make some material (just the script used, page 24–26) available for download together with a tutorial, best of course integrated into R or some other gene expression analysis standard.

Authors’ response: *The code is currently available at the supporting data website at* http://mendel.bii.a-star.edu.sg/SEQUENCES/PLSDBC/*, but it is likely that we will re-write the code in R language and to provide tutorial for future releases.*

6) General final comment: the better understandable the language, the easier and intuitive clear figures, the more the people will understand your nice findings and actually APPLY them (which currently does not happen so often and hence leads then to wrong conclusions).

Authors’ response: *We thank the reviewer for his positive comments of our work and his constructive suggestions to improve this manuscript.*

### Reviewer’s report 3: Sandor Pongor, International Centre for Genetic Engineering and Biotechnology (ICGEB), Italy

To the discretion of the authors: The authors may want to show results on more datasets or just preliminarily indicate how the findings generalize to other datasets. Also, instructions for practical use, availability of codes would be useful provided the authors do not plan to publish these data elsewhere.

Authors’ response: *We thank the reviewer for his positive comments. In fact, the concept has been generalized beyond microRNA to messenger RNA sequencing data (see* Additional file 9: Fig. S6*) where we found the general trend of Zipf’s law in transcript abundance. In an on-going work, we were able to show an increase in sensitivity of a miRNA-mRNA analysis that leads to enhanced biological conclusion when the finite-size effect or power-law correction is applied; This is in a current working manuscript.*

*Also, although the code is already currently available at the supporting data website* i.e. http://mendel.bii.a-star.edu.sg/SEQUENCES/PLSDBC/*, it is likely that we will re-write the code in R language for future releases.*

## Additional files

Additional file1: Figure S1. Concentration versus total mapped reads of the dilution data set. Figures S1A to D shows the concentrations of the AGS cell line (*12pM, 3pM*) and NUGC3 cell line (*12pM, 6pM, 3pM, 1.5pM*) versus the respective total mapped reads by the 4 mapping methods: Bowtie1, Bowtie2(global), Novoalign and BWA. Regardless of the mapping methods, the sequencing depth (i.e.*, the total mapped reads*) is shown to be linearly proportional to the system size (*in terms of transcript concentration*) in the logarithmic scale. Overall, the dilution data set attempts to mimic a system of various sizes of finite-size effects. (PNG 371 kb)

Additional file 2: Figure S2. Pareto distributions and scatterplots of spike-in background data set. Figure S2A to F show the Pareto plots (*left column*) and supplementary Figure S2G to L show the scatterplots (*right column*) of the spike-in background set where each applied normalization methods (i.e.*, DESeq, RLE, TMM, UQ, CPM and Quantile*) are arranged row-wise. Generally speaking, the characteristics of these Pareto plots of the normalized spike-in background set are very comparable to that of Fig. 1A and C, where only a simple intra-sample scaling has been applied. Despite the application of normalization, two characteristics remain unchanged. Firstly, the non-uniform slope values and its decreasing trend from the highest to lowest-count segment indicate that heteroskedasticity among the replicates will remain. Secondly, for those count segment with slope values far from “-1”, their mathematical moments are infinite and hence, large variation among the replicates will be expected for these segments. (PNG 1662 kb)

Additional file 3: Figure S3. Pareto distributions and scatterplots of NUGC3 dilution data set. Figures S3A to F show the Pareto plots (*left column*) and Figure S3G to L show the scatterplots (*right column*) of the spike-in background set where each applied normalization methods (i.e.*, DESeq, RLE, TMM, UQ, CPM and Quantile*) are arranged row-wise. Likewise, the same conclusion can be made of the Pareto and scatterplots of the NUGC3 dilution set (Fig. 3A-F) versus Fig. 1B and D where both the exaggerated spit-end among the Pareto plots and the extreme heteroskedasticity of the scatter plots in the NUGC3 dilution set remain. (PNG 1415 kb)

Additional file 4: Table S1. Signal-to-noise characteristics of the comparative dilution analysis (AGS versus NUGC3) before and after power-law correction. (DOCX 21 kb)

Additional file 5: Table S2. Significant transcripts calls of comparative dilution analysis (AGS versus NUGC3) before and after power-law correction. (DOCX 16 kb)

Additional file 6: Figure S5. Medians of Regressed slopes of first-fitted segment versus R^2^ fit for the full dilution and spike-in datasets. Figure S5A and 5B show the median slope of the first-fitted segments versus the median R^2^ value of the dilution and the spike-in data set respectively. In both plots, the refined solution space of the optimum points-per-segment (PPS; as indicated besides the data points) is indicated by the error margins defined by the slope of the first highest-count segments from Table 1 like before. Consequently, the optimum PPS value is determined by the largest average R^2^ value where it is 55 for the dilution set and 10 for the spike-in set. Note that due to the lack of replicates for the spike-in transcripts, only the background of the spike-in set was used for the parameter estimation. (PNG 315 kb)

Additional file 7: Table S3. Concordance list of miRNA transcripts before and after power-law correction. (DOCX 12 kb)

Additional file 8: Figure S4. Medians of Regressed slopes of first-fitted segment versus R^2^ fit for NUGC3 dilution and spike-in background datasets. Figure S4A and 4B show the median slope of the first-fitted segments versus the median R^2^ value of the spike-in background set and the NUGC3 dilution set respectively. For the necessary R^2^ computations, the reference replicate was taken as the replicate with the largest total reads within the data series. In both plots, the refined solution space of the optimum points-per-segment (PPS; as indicated besides the data points) is indicated by the error margins defined by the slope of the first highest-count segments from Table 1. Within this margin, the optimum PPS value is determined by the largest average R^2^ value where it is 20 for the spike-in background set and 45 for the NUGC3 dilution data sets. (PNG 316 kb)

Additional file 9: Figure S6. Pareto distributions of Universal Human Reference (UHR) mRNA HTSeq-mapped and RSEM-mapped sequencing count data. Figures S6A and B show the Pareto distributions of the Universal Human Reference (UHR) mRNA data set from the publicly available source - GSE47774 that has been quantified by HTSeq and RSEM respectively. Generally, Zipf’s law holds approximately for the middle segments of the observed distributions despite the differences in abundance quantification approach between HTSeq [43] and RSEM [44]; HTSeq tends to be more conservative than RSEM by limiting quantification to uniquely mapped reads. Meanwhile, the low abundance segments exhibit different trends. Of particular interest is that the highest and high segment in NGS-based mRNA data seems to exhibit a higher slope than the Zipf’s law that characterized SAGE-based mRNA data. Preliminary findings suggests that this might be attributed to transcript-length bias in NGS-basedsequencing that is absent in SAGE-based sequencing [9]. Nevertheless, Type I Pareto distribution (or approximately Zip’s law) seemingly holds true for transcript abundance distributions despite the differences in technology (SAGE versus NGS) and RNA species (miRNA and mRNA). (PNG 313 kb)

## Abbreviations

CDF: Cumulative density function
CPM: Count Per Million
CTF: Cumulative total function
NGS: Next-generation sequencing
PDF: Probability density function
RLE: Relative Log Expression
SNR: Signal-to-noise ratio
TMM: Trimmed Mean of M-values
UQ: UpperQuartile
