# Supplementary material for: Finite-size effects in transcript sequencing count distribution: its power-law correction necessarily precedes downstream normalization and comparative analysis
Source: Biol Direct. 2018 Feb 12;13:2. doi: 10.1186/s13062-018-0204-y (PMC5809866; doi:10.1186/s13062-018-0204-y)
Supplement: Supplementary file 4 — Signal-to-noise characteristics of the comparative dilution analysis (AGS versus NUGC3) before and after power-law correction. (DOCX 21 kb) [file 13062_2018_204_MOESM4_ESM.docx]

**Supplementary Table 1**

**Signal‑to‑noise characteristics of the comparative dilution analysis (AGS versus NUGC3) before and after power‑law correction**

This table complements the MA‑plots in Figures 6A to D. It summarizes the characteristics of the signal and noise comparisons before and after power‑law correction for each aligner across 6 normalization methods. The bias and variance of each normalization method, in terms of signal and noise, are computed from the difference between the comparisons and the fitted noise model and with the summary statistics taken. The signal‑to‑noise ratio, before and after power‑law correction, are also given. The average signal‑to‑noise ratio improvement is about 1.5 times after the correction.

|  |  | **Original data** | | | **Power‑law corrected data** | | |
| --- | --- | --- | --- | --- | --- | --- | --- |
| **Mapping method** | **Normalization method** | **Residual**  **(µ±σ)_noise_** | **Residual**  **(µ±σ)_signal_** | **Signal-to-noise ratio $\frac{E(x_{signal}^{2})}{\sigma_{noise}^{2}}$** | **Residual**  **(µ±σ)_noise_** | **Residual**  **(µ±σ)_signal_** | **Signal-to-noise ratio**  **$\frac{E(x_{signal}^{2})}{\sigma_{noise}^{2}}$** |
| Bowtie1 | DESeq | 0.019±0.662 | -0.853±2.240 | 9.5 | 0.004±0.274 | 0.027±1.021 | 14.3 |
|  | RLE | 0.005±0.629 | 0.047±2.206 | 12.4 | -0.003±0.248 | 0.013±1.013 | 16.9 |
|  | TMM | 0.019±0.637 | -0.122±2.218 | 11.8 | 0.001±0.250 | -0.107±1.012 | 14.7 |
|  | UQ | 0.016±0.675 | -0.262±2.278 | 10.8 | 0.003±0.286 | -0.107±1.031 | 11.7 |
|  | CPM | 0.022±0.670 | -0.863±2.256 | 9.4 | 0.003±0.272 | 0.028±1.021 | 14.5 |
|  | Quantile | 0.001±0.555 | -0.002±2.165 | 15.2 | 0.000±0.201 | -0.001±1.033 | 26.4 |
|  | median of all | 0.018±0.649 | -0.192±2.229 | 11.3 | 0.002±0.261 | 0.006±1.021 | 15.4 |
| Bowtie2 (global) | DESeq | 0.022±0.655 | -0.872±2.211 | 9.4 | 0.002±0.253 | 0.052±1.022 | 17.1 |
|  | RLE | 0.008±0.620 | 0.058±2.177 | 12.5 | -0.003±0.235 | 0.009±1.012 | 18.7 |
|  | TMM | 0.022±0.629 | -0.099±2.188 | 11.9 | 0.000±0.236 | -0.132±1.012 | 16.0 |
|  | UQ | 0.017±0.678 | -0.240±2.249 | 10.5 | 0.002±0.271 | -0.095±1.036 | 13.4 |
|  | CPM | 0.025±0.663 | -0.880±2.227 | 9.3 | 0.002±0.251 | 0.053±1.022 | 17.3 |
|  | Quantile | 0.007±0.539 | -0.004±2.127 | 15.6 | 0.001±0.199 | -0.002±1.024 | 26.5 |
|  | median of all | 0.019±0.642 | -0.169±2.200 | 11.3 | 0.002±0.244 | 0.003±1.022 | 17.6 |
| Novoalign | DESeq | 0.022±0.653 | -0.900±2.200 | 9.2 | 0.002±0.248 | 0.067±1.017 | 17.9 |
|  | RLE | 0.008±0.618 | 0.049±2.165 | 12.4 | -0.002±0.230 | 0.001±1.007 | 19.2 |
|  | TMM | 0.022±0.629 | -0.116±2.178 | 11.7 | -0.002±0.229 | -0.061±1.006 | 18.1 |
|  | UQ | 0.013±0.698 | -0.191±2.245 | 10.0 | 0.002±0.276 | -0.088±1.033 | 12.9 |
|  | CPM | 0.024±0.662 | -0.911±2.216 | 9.1 | 0.002±0.246 | 0.069±1.016 | 18.2 |
|  | Quantile | 0.009±0.534 | -0.005±2.117 | 15.7 | 0.001±0.194 | -0.002±1.021 | 27.7 |
|  | median of all | 0.017±0.641 | -0.153±2.189 | 11.3 | 0.001±0.238 | -0.001±1.017 | 18.2 |
| BWA | DESeq | 0.021±0.660 | -0.896±2.203 | 9.1 | 0.002±0.252 | 0.057±1.019 | 17.3 |
|  | RLE | 0.007±0.625 | 0.052±2.170 | 12.2 | -0.003±0.234 | 0.003±1.011 | 18.8 |
|  | TMM | 0.021±0.636 | -0.097±2.182 | 11.6 | -0.001±0.234 | -0.103±1.009 | 16.8 |
|  | UQ | 0.012±0.701 | -0.221±2.247 | 9.8 | 0.002±0.282 | -0.096±1.034 | 12.2 |
|  | CPM | 0.023±0.667 | -0.906±2.219 | 9.0 | 0.002±0.250 | 0.057±1.019 | 17.5 |
|  | Quantile | 0.005±0.543 | -0.006±2.121 | 15.2 | 0.001±0.199 | -0.002±1.023 | 26.3 |
|  | median of all | 0.017±0.648 | -0.159±2.193 | 11.1 | 0.001±0.242 | 0.001±1.019 | 17.8 |
