# Supplementary material for: Finite-size effects in transcript sequencing count distribution: its power-law correction necessarily precedes downstream normalization and comparative analysis
Source: Biol Direct. 2018 Feb 12;13:2. doi: 10.1186/s13062-018-0204-y (PMC5809866; doi:10.1186/s13062-018-0204-y)
Supplement: Supplementary file 5 — Significant transcripts calls of comparative dilution analysis (AGS versus NUGC3) before and after power-law correction. (DOCX 16 kb) [file 13062_2018_204_MOESM5_ESM.docx]

**Supplementary Table 2**

**Significant transcripts calls of comparative dilution analysis (AGS versus NUGC3) before and after power‑law correction**

The breakdown of significant transcript calls for each combination of the mapping algorithms (*Bowtie1, Bowtie2(global), Novoalign and BWA*) and normalization methods (*DESeq, RLE, TMM, Upperquartile, CPM and Quantile*) for all 4 positive comparisons (*AGS‑12p versus NUGC‑12p, AGS‑12p versus NUGC‑3p, AGS‑3p versus NUGC‑12p and AGS‑3p versus NUGC‑3p*) are given in the following table. The median number of significant calls for 6 normalization methods are highlighted in red for each mapping algorithm.

|  |  | **Original data** | | | | **Power‑law corrected data** | | | |
| --- | --- | --- | --- | --- | --- | --- | --- | --- | --- |
| **Mapping**  **Method** | **Normalization method** | **AGS 12p**  **vs**  **NUGC3 12p** | **AGS 12p**  **vs**  **NUGC3 3p** | **AGS 3p**  **vs**  **NUGC3 12p** | **AGS 3p**  **vs**  **NUGC3 3p** | **AGS 12p**  **vs**  **NUGC3 12p** | **AGS 12p**  **vs**  **NUGC3 3p** | **AGS 3p**  **vs**  **NUGC3 12p** | **AGS 3p**  **vs**  **NUGC3 3p** |
| Bowtie1 | DESeq | 31 | 30 | 28 | 28 | 55 | 48 | 51 | 46 |
|  | RLE | 59 | 58 | 51 | 43 | 61 | 55 | 64 | 56 |
|  | TMM | 42 | 39 | 38 | 34 | 60 | 52 | 61 | 54 |
|  | UQ | 48 | 42 | 40 | 37 | 56 | 52 | 47 | 43 |
|  | CPM | 40 | 29 | 28 | 24 | 57 | 50 | 50 | 44 |
|  | Quantile | 42 | 42 | 43 | 42 | 54 | 54 | 52 | 53 |
|  | Median of all | 42 | 41 | 39 | 36 | 57 | 52 | 52 | 50 |
| Bowtie2  (global) | DESeq | 41 | 36 | 34 | 32 | 60 | 59 | 58 | 56 |
|  | RLE | 65 | 70 | 57 | 46 | 73 | 66 | 83 | 69 |
|  | TMM | 53 | 48 | 48 | 44 | 68 | 65 | 75 | 70 |
|  | UQ | 43 | 43 | 39 | 37 | 58 | 57 | 54 | 50 |
|  | CPM | 42 | 37 | 33 | 28 | 61 | 59 | 58 | 53 |
|  | Quantile | 45 | 43 | 46 | 45 | 58 | 56 | 64 | 59 |
|  | Median of all | 44 | 43 | 43 | 41 | 61 | 59 | 61 | 58 |
| Novoalign | DESeq | 36 | 32 | 30 | 29 | 57 | 53 | 54 | 51 |
|  | RLE | 65 | 69 | 58 | 44 | 68 | 61 | 71 | 63 |
|  | TMM | 45 | 45 | 43 | 36 | 66 | 60 | 66 | 58 |
|  | UQ | 50 | 44 | 40 | 38 | 57 | 57 | 49 | 48 |
|  | CPM | 41 | 32 | 31 | 27 | 58 | 53 | 51 | 48 |
|  | Quantile | 35 | 35 | 37 | 35 | 58 | 56 | 59 | 57 |
|  | Median of all | 43 | 40 | 39 | 36 | 58 | 57 | 57 | 54 |
| BWA | DESeq | 34 | 33 | 28 | 28 | 57 | 53 | 55 | 50 |
|  | RLE | 66 | 71 | 59 | 47 | 64 | 55 | 68 | 57 |
|  | TMM | 42 | 44 | 42 | 35 | 65 | 59 | 68 | 59 |
|  | UQ | 45 | 41 | 36 | 36 | 56 | 55 | 52 | 48 |
|  | CPM | 40 | 33 | 28 | 26 | 58 | 53 | 53 | 50 |
|  | Quantile | 39 | 40 | 41 | 41 | 56 | 55 | 56 | 55 |
|  | Median of all | 41 | 41 | 39 | 36 | 58 | 55 | 56 | 53 |
