# Supplementary material for: Finite-size effects in transcript sequencing count distribution: its power-law correction necessarily precedes downstream normalization and comparative analysis
Source: Biol Direct. 2018 Feb 12;13:2. doi: 10.1186/s13062-018-0204-y (PMC5809866; doi:10.1186/s13062-018-0204-y)
Supplement: Supplementary file 7 — Concordance list of miRNA transcripts before and after power-law correction. (DOCX 12 kb) [file 13062_2018_204_MOESM7_ESM.docx]

**Supplementary Table 3**

**Concordance list of miRNA transcripts before and after power‑law correction**

Based on the Bowtie1‑mapped results from Table 5, the concordance transcripts across the 4 comparisons before (*see "intersect row"; columns 3‑6*) and after power‑law (*see "intersect row"; columns 7‑10*) correction were compiled into a union set of concordance transcripts. This gives a total of 30 (*column 1*) and 52 (*column 2*) concordance pre‑cursor miRNA transcripts respectively. Due to slight variations among the 4 comparisons, the uncorrected list exceeded the maximum intersect value of 28 (*AGS‑12p versus NUGC3‑12p*). In addition, the lists of miRNA transcripts that are unique to the uncorrected (*column 3*) and power‑corrected (*column 4*) analysis are also listed.

| **Original data**  **(30 transcripts)** | **PL‑corrected data**  **(52 transcripts)** | **Unique to Original data (1 transcript)** | **Unique to PL‑corrected data (23 transcripts)** |
| --- | --- | --- | --- |
| hsa-mir-100  hsa-mir-126  hsa-mir-130a  hsa-mir-146a  hsa-mir-148a  hsa-mir-181a-1  hsa-mir-192  hsa-mir-194-1  hsa-mir-194-2  hsa-mir-196a-1  hsa-mir-196a-2  hsa-mir-203a  hsa-mir-203b  hsa-mir-205  hsa-mir-215  hsa-mir-222  hsa-mir-30a  hsa-mir-335  hsa-mir-371a  hsa-mir-371b  hsa-mir-372  hsa-mir-373  hsa-mir-508  hsa-mir-509-1  hsa-mir-509-2  hsa-mir-509-3  hsa-mir-577  hsa-mir-584  hsa-mir-767  hsa-mir-9-1 | hsa-let-7a-1  hsa-let-7a-2  hsa-let-7a-3  hsa-let-7e  hsa-let-7i  hsa-mir-100  hsa-mir-10a  hsa-mir-126  hsa-mir-130a  hsa-mir-146a  hsa-mir-148a  hsa-mir-16-1  hsa-mir-16-2  hsa-mir-181a-1  hsa-mir-181a-2  hsa-mir-181b-1  hsa-mir-181b-2  hsa-mir-181c  hsa-mir-191  hsa-mir-192  hsa-mir-194-1  hsa-mir-194-2  hsa-mir-196a-1  hsa-mir-196a-2  hsa-mir-203a  hsa-mir-203b  hsa-mir-205  hsa-mir-218-2  hsa-mir-221  hsa-mir-222  hsa-mir-27b  hsa-mir-29a  hsa-mir-30a  hsa-mir-30d  hsa-mir-31  hsa-mir-335  hsa-mir-371a  hsa-mir-371b  hsa-mir-372  hsa-mir-373  hsa-mir-508  hsa-mir-509-1  hsa-mir-509-2  hsa-mir-509-3  hsa-mir-577  hsa-mir-584  hsa-mir-767  hsa-mir-9-1  hsa-mir-9-2  hsa-mir-92a-1  hsa-mir-92a-2  hsa-mir-98 | hsa-mir-215 | hsa-let-7a-1  hsa-let-7a-2  hsa-let-7a-3  hsa-let-7e  hsa-let-7i  hsa-mir-10a  hsa-mir-16-1  hsa-mir-16-2  hsa-mir-181a-2  hsa-mir-181b-1  hsa-mir-181b-2  hsa-mir-181c  hsa-mir-191  hsa-mir-218-2  hsa-mir-221  hsa-mir-27b  hsa-mir-29a  hsa-mir-30d  hsa-mir-31  hsa-mir-9-2  hsa-mir-92a-1  hsa-mir-92a-2  hsa-mir-98 |
