# Supplementary figures and images for: Finite-size effects in transcript sequencing count distribution: its power-law correction necessarily precedes downstream normalization and comparative analysis
Source: Biol Direct. 2018 Feb 12;13:2. doi: 10.1186/s13062-018-0204-y (PMC5809866; doi:10.1186/s13062-018-0204-y)

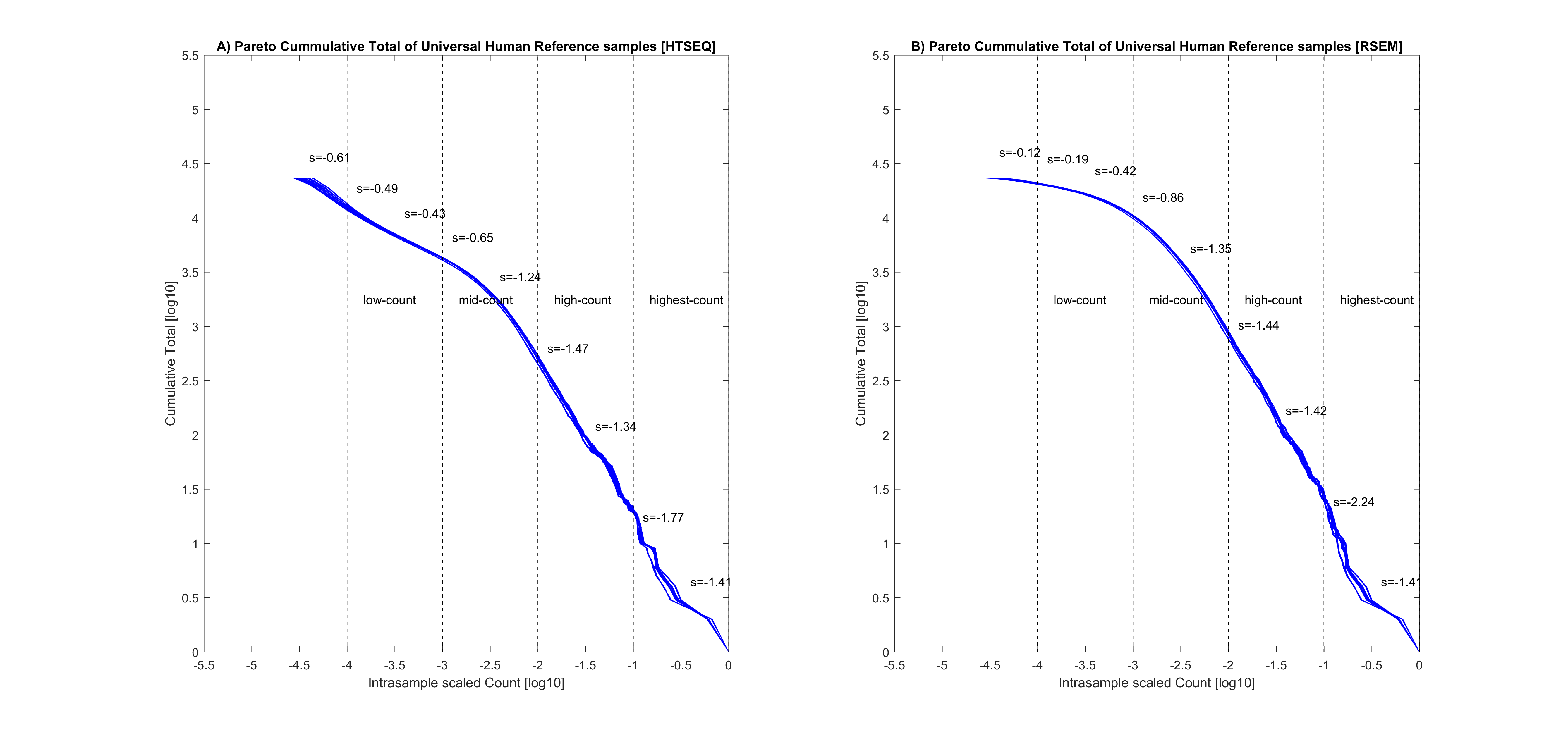

Supplement: Supplementary file 9 — Pareto distributions of Universal Human Reference (UHR) mRNA HTSeq-mapped and RSEM-mapped sequencing count data. Figures S6A and B show the Pareto distributions of the Universal Human Reference (UHR) mRNA data set from the publicly available source - GSE47774 that has been quantified by HTSeq and RSEM respectively. Generally, Zipf’s law holds approximately for the middle segments of the observed distributions despite the differences in abundance quantification approach between HTSeq [43] and RSEM [44]; HTSeq tends to be more conservative than RSEM by limiting quantification to uniquely mapped reads. Meanwhile, the low abundance segments exhibit different trends. Of particular interest is that the highest and high segment in NGS-based mRNA data seems to exhibit a higher slope than the Zipf’s law that characterized SAGE-based mRNA data. Preliminary findings suggests that this might be attributed to transcript-length bias in NGS-basedsequencing that is absent in SAGE-based sequencing [9]. Nevertheless, Type I Pareto distribution (or approximately Zip’s law) seemingly holds true for transcript abundance distributions despite the differences in technology (SAGE versus NGS) and RNA species (miRNA and mRNA). (PNG 313 kb) [file 13062_2018_204_MOESM9_ESM.png]
